# Correction: Decentralized control of insect walking: A simple neural network explains a wide range of behavioral and neurophysiological results

**DOI:** 10.1371/journal.pcbi.1009362

**Published:** 2021-09-02

**Authors:** The PLOS Computational Biology Staff

Several errors were introduced during the production of this article. The publisher apologizes for the errors.

In the Discussion, there is an error in the sixth sentence of the second paragraph. The correct sentence is: Patterns recently described as non-canonical by DeAngelis et al. [50] can be observed, too (e.g., S2B Fig, S2C Fig).

In the B) Leg control. sub-subsection of The neural Controller subsection of the Methods, there is an error in the second sentence of the ninth paragraph. The correct sentence is: An error signal is calculated from the currently sensed joint position (given by the black squares addressed above) and a set point provided as a target position for the swing movement (set points may vary during negotiation of curves, see below).

In the C) Interleg coordination. sub-subsection of The neural Controller subsection of the Methods, there is an error in the third sentence of the first paragraph. The correct sentence is: Here we deal with rules 1–3 first (all units marked brown, Fig 2; these have been realized in Walknet as well [23]) and later rule 5 (all units marked “ocher”, Fig 2; this has been realized in an earlier version of Walknet [86]).

Fig 10 and Fig 12 appear incorrectly. Fig 10 appears with incorrect dimensions due to a production error. In Fig 12, the connection between L3 and R3 should read: 2,3,(5?). Please view the correct Figs 10 and 12 below.

**Fig 10:**
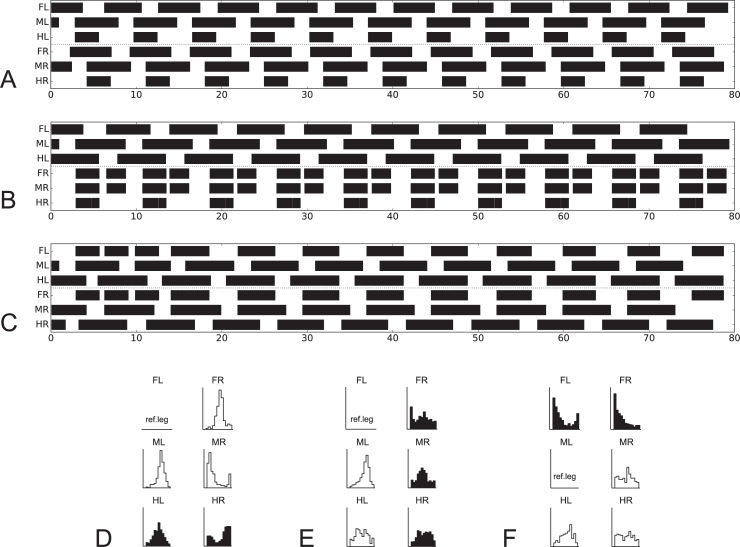
Simulation of experiments where stick insects walk tethered on a treadmill with selected legs standing on force transducer platforms. Shown are activations of retractor motor neurons (> 7 mV) over time (s) in A) to C). Corresponding experimental results [76,86] are given in D, E, F, respectively: Phase histograms of the beginning of the retraction in the walking legs (shown in white), force histograms of the maximum force in the standing legs (dark). Reference leg starting with the beginning of the retraction movement. In A) and D) both front legs and both middle legs are walking while both hind legs are standing. B) and E) shows left legs walking and right legs standing. C) and F) show both front legs standing, both middle legs and both hind legs walking [76,86]. Walking velocity neurons set to 30 mV.

**Fig 12:**
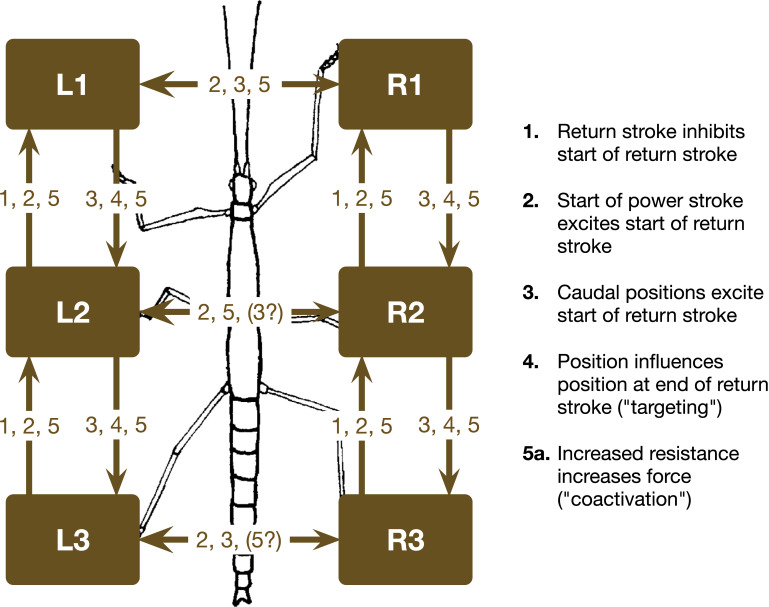
Single leg controllers (shown in brown) and their connection via coordination rules (from [15]). L1, L2, L3 left front, middle, and hind leg, respectively. R1, R2, and R3 stand for the corresponding right legs. The question mark indicates that there are ambiguous data concerning influence 3.

S1 Document, S2 Document, and S3 Document contain incorrect heading numbers. Please view the correct S1-S3 Documents below.

## Supporting information

S1 Document Sections of steps for different legs. (DOCX)

S2 Document Results and adaptation of model for running. (DOCX)

S3 Document Differences between species concerning interleg coordination and chosen coordination influences. (DOCX)
