# Supplementary material for: Decentralized control of insect walking: A simple neural network explains a wide range of behavioral and neurophysiological results
Source: PLoS Comput Biol. 2020 Apr 27;16(4):e1007804. doi: 10.1371/journal.pcbi.1007804 (PMC7205325; doi:10.1371/journal.pcbi.1007804)
Supplement: S2 Document — (PDF) [file pcbi.1007804.s006.pdf]

Supporting Information

# S6 – **Running**

in Malte Schilling and Holk Cruse (2020): Decentralized control of insect walking - a simple neural network explains a wide range of behavioral and neurophysiological results. *PLOS Computational Biology*

While neuroWalknet can serve as a hypothesis describing the control of hexapod walking covering very slow ("pentapod"), medium ("tetrapod") and fast ("tripod") walking, there are basic open questions concerning control of running. How might a controller allowing for running have been developed? In walking insects, this faculty appears to be only used when the legs are under high load [1], but not during "normal" walking anymore.

The faculty of running (beyond step frequencies of about 5 steps/s) as observed in cockroaches may represent the result of a subsequent evolutionary development, following the constraint that running does not provide enough time required for sensory feedback. As addressed in the Introduction and the Discussion, for running the sensory delay does not enable control of walking based on sensory feedback beyond a given velocity. For insects of the size of cockroaches as Periplaneta americana, or Blaberus discoidalis this limit is estimated to exist at about 5 - 7 steps /s [2–4]. Therefore, our network being based on sensory feedback cannot simply be sped up to higher velocities, if the biological conditions should be addressed. The generally proposed solution to this problem is to use CPGs for controlling the rhythmic movements (see however Introduction, [5], Sasha Zill, pers. communication). In the following we show how, using a minimal expansion of neuroWalknet, properties of CPGs might arise and how the two basic problems, intraleg coordination and interleg coordination, might be solved.

First, we introduced a motivation unit (see Conclusions) that when activated stabilizes the network in state "Run". Introduction of such a separate state for running may be justified because specific ("fast") muscles are activated only when step frequency goes beyond the limit of 5 steps/s [6]. The WTA networks used in slow walking to avoid cocontraction (Fig. 2, PMN) are, in state "Run", used to control the motor output for fast walking. Second, motivation unit "Run" activates a small network ("SRP") that connects the joints within a leg. Third, unit "Run" activates a network similar to the rule 5 connections allowing for a stable tripod-like gait.

Concerning the CPGs, we simply use the WTA networks given (Fig. 2, red units, PMN). These are now actuated by an input from the motivation unit "Run" (to keep Fig. 2 as simple as possible we did not depict this unit) instead of the pilocarpine activation (input "pilo) that was exciting the sensory nerves in deafferented preparations. In addition, the appropriate oscillating frequency and the temporal ratio of the antagonist activity could be controlled by influencing the relaxation time constant of the inhibitory units of the corresponding WTA net (red units, PMN, Fig. 2). This may be realized by using input from the unit "Run" to threshold the excitation of the inhibitory units of the WTA nets. However, as a proof of concept we will deal with one velocity value only. Therefore, we chose values that provide a ratio of duration of about 1:3 for levator – depressor, and for extensor – flexor pairs and of about 2:2 for the protractor – retractor pair.

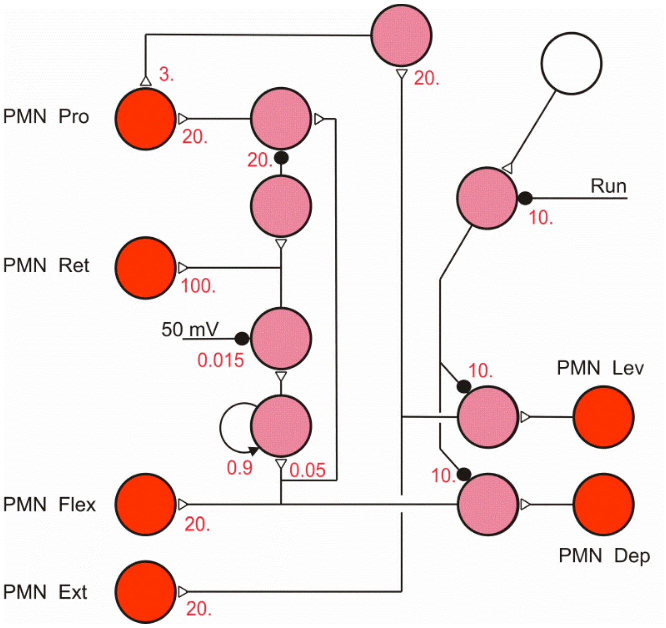


**Figure S5A. SRP-net,** used for intraleg coordination during running (see Fig. 2, pink triangle). PMN: premotor units (red), alpha joint: protractor, retractor; beta joint: levator, depressor; gamma joint: flexor, extensor, as depicted in Fig. 2. White unit is continuously activated (50 mV).

To couple the three joints of a leg, we introduce a small network termed spontaneous recurrent patterns (SRP)-net, following results of Büschges et al. [7]. These authors observed in deafferented stick insects an often appearing temporal relation between a switch from levator activity to depressor activity being accompanied by a stop of extensor activity, which was followed, after a short period, by a switch from protractor to retractor activity. We interpret this observation in such a way that connections exist from depressor to flexor, to protractor and, delayed, to retractor as well as from levator to protractor and extensor. This hypothetical network is depicted by a triangular pink box in Fig. 2. The complete network is given in Fig. S5A. Functionally, this network activates levator, protractor and extensor at a moment corresponding to PEP in slow walking, and activates depressor at the upper extreme position during swing, and retractor at a moment corresponding to AEP in slow walking. Note that this is different to earlier approaches that introduced intraleg coupling using sensory feedback (e.g. [8,9]).

To address the question concerning interleg coupling, we applied a network (Fig. 2, light yellow units) very similar to that used for rule 5, but introduce, based on results of Pearson and Iles [10], inhibitory influences from a levator unit to the levators of the directly neighboring legs instead of positive feedback as used in the case of rule 5. These 'Pearson-rule' connections lead to a phase distribution in such a way that a levator burst is either directly followed or directly preceded by the levator burst of its neighboring leg ([10], their Fig. 4).

In the simulation, all sensory inputs are suppressed by means of the motivation unit "Run" to simulate the situation where appropriate sensory input is not available. Note that in this simulation interleg coordination relies on mutual inhibition between all directly neighboring legs, whereas for intraleg coordination a feedforward net is used where the levator – depressor CPG controls the other joints. The units forming the bistable monopole controlling swing and stance (light grey units, Fig. 2) are not activated in this mode.

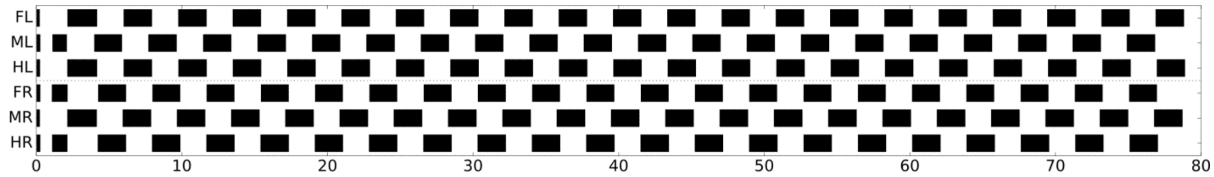


**Figure S5B. Footfall pattern for running.** Running is illustrated by activity of protractor output (> 0 mV), therefore looking similar to footfall pattern as shown for earlier slow, middle and fast walking examples. Ordinate: legs as in Fig. 3, abscissa: time (s).

For testing a solution using minimal feedback we did not influence the CPGs as such, but introduced one position threshold for each joint in a way that the motor output of the joint was set to zero velocity if this threshold has been reached. Application of an at least one-sided threshold allows stable walking patterns, a tripod pattern or possibly some kind of mixture between tripod and tetrapod pattern [11], resulting from the limitations given by the Pearson rule. An example is given in Fig. S5B (+ video) showing a tripod-like case. Note that this pattern (Fig. S5B) shows activation of protractor output and is therefore similar, but not identical to the footfall patterns showed in earlier figures (showing activation of swing state). We tested different starting positions, but did not perform more detailed investigation concerning the stability of the pattern by using systematic disturbances as has been done for walking.

If only the extreme mechanical limits (i.e. position 0 mV and 50 mV) are given as a threshold, but velocity output to the motors is not switched off, stable patterns can still be observed when focusing on the temporal pattern of the motor outputs. However, to reach cyclic movements of the legs themselves, one has to compensate for possible drift of leg positions. The thresholds required could be realized through nonlinear properties of the muscles for example [12].

In deafferented cockroaches that were treated with pilocarpine, an anti-phase, tripod-like pattern has been observed [13]. This is in contrast to in-phase coupling found in deafferented locusts, stick insects, rock lobster and crayfish as mentioned earlier. Correspondingly, experiments with intact walking legs and deafferented neighboring legs as performed by Borgmann et al. [14] with stick insects and by Clarac and Chasserat [15] with crayfish showed in-phase coupling, whereas cockroaches revealed an anti-phase coupling in this situation. These results (Fuchs et al., 2011) could be simulated, too, if we instead of rule 5 now assume that ‘Pearson-rule’ network is activated. Interestingly, a change from in-phase coupling to anti-phase coupling between neighboring legs has been observed in *Manduca sexta* during metamorphosis [16], which may be interpreted as ontogenetic recapitulation of a phylogenetic development.

Taken together, we suggest that running may have been developed beyond the ability of slow and fast walking by minor changes of already existing neural systems. For example, CPG properties are gained by common input to both PMNs controlling a joint, whereas the “Pearson-rule” network may have been evolved through a minor change of the rule 5 connectivity. The question whether rhythmic movement is controlled via “minimal neural feedback” or specific nonlinear properties of muscles (“preflexes”) that allow for self-stabilization (e.g. [17,18]) is still open. Near the upper end of the speed limit, different species appear to have found evolutionary solutions that seem to differ in detail (see Weihmann et al. [19] for a detailed discussion).

## References


1. Cruse H. Coactivating influences between neighbouring legs in walking insects. Journal of Experimental Biology. 1985;114:513 519.

2. Delcomyn F. Perturbation of the motor system in freely walking cockroaches. I. Rear leg amputation and the timing of motor activity in leg muscles. Journal of Experimental Biology. 1991;156:483–502.

3. Sponberg S, Full RJ. Neuromechanical response of musculo-skeletal structures in cockroaches during rapid running on rough terrain. J Exp Biol. 2008 Feb;211(Pt 3):433–46.

4. Zill SN, Moran DT. The Exoskeleton and Insect Proprioception: III. Activity of Tribal Campaniform Sensilla During Walking in the American Cockroach, Periplaneta Americana. Journal of Experimental Biology. 1981 Oct 1;94(1):57–75.

5. Höltje M, Hustert R. Rapid mechano-sensory pathways code leg impact and elicit very rapid reflexes in insects. J Exp Biol. 2003;206:2713–2724,.

6. Watson JT, Ritzmann RE. Leg kinematics and muscle activity during treadmill running in the cockroach, Blaberus discoidalis: I. Slow running. Journal of Comparative Physiology. 1997;

7. Büschges A, Schmitz J, Bässler U. Rhythmic patterns in the thoracic nerve cord of the stick insect induced by pilocarpine. J Exp Biol. 1995;198,435-456.

8. Büschges A. Lessons for circuit function from large insect: towards understanding the neural basis of motor flexibility. Current Opinion in Neurobiology. 2012;22:602–608.

9. Tóth TI, Daun S. A kinematic model of stick-insect walking. Physiological Reports [Internet]. 2019 Apr 1 [cited 2019 May 2];7(8). Available from: https://physoc.onlinelibrary.wiley.com/doi/abs/10.14814/phy2.14080

10. Pearson KG, Iles JF. Nervous mechanisms underlying intersegmental co-ordination of the leg movements during walking in the cockroach. Journal of Experimental Biology. 1973;58:725–744.

11. Weihmann T, Brun PG, Pycroft E. Speed dependent phase shifts and gait changes in cockroaches running on substrates of different slipperiness. Frontiers in Zoology. 2017;14(54).

12. Kukillaya R, Holmes P. A hexapedal jointed-leg model for insect locomotion in the horizontal plane. Biological Cybernetics. 2007 Dec;97(5–6):379--395.

13. Fuchs E, Holmes P, Kiemel T, Ayali A. Intersegmental coordination of cockroach locomotion: adaptive control of centrally coupled pattern generator circuits. Frontiers in neural circuits. 2011;4.

14. Borgmann A, Hooper SL, Büschges A. Sensory Feedback Induced by Front-Leg Stepping Entrains the Activity of Central Pattern Generators in Caudal Segments of the Stick Insect Walking System. The Journal of Neuroscience. 2009;29(9).

15. Clarac F, Chasserat C. Experimental modification of interlimb coordination during locomotion of a Crustacea. Neuroscience letters. 1979;12:271–6.

16. Johnston RM, Levine RB. Thoracic leg motoneurons in the isolated CNS of adult Manduca produce patterned activity in response to pilocarpine, which is distinct from that produced in larvae. Invert Neurosc. 2002;4:175–192.

17. Jindrich DL, Full RJ. Dynamic stabilization of rapid hexapedal locomotion. J Exp Biol. 2002 Sep;205(Pt 18):2803–23.

18. Koditschek DE, Full RJ, Buehler M. Mechanical aspects of legged locomotion control. Arthropod Struct Dev. 2004;33:251–257.

19. Weihmann T, Goetzke HH, Günther M. Requirements and limits of anatomy-based predictions of locomotion in terrestrial arthropods with emphasis on arachnids. J Paleontol. 2015;89:980–90.
