# Supplementary material for: Decentralized control of insect walking: A simple neural network explains a wide range of behavioral and neurophysiological results
Source: PLoS Comput Biol. 2020 Apr 27;16(4):e1007804. doi: 10.1371/journal.pcbi.1007804 (PMC7205325; doi:10.1371/journal.pcbi.1007804)
Supplement: Attachment — Submitted filename: schilling_PCOMPBIOL-D-19-01265R1_reply_reviewer.pdf [file pcbi.1007804.s023.pdf]

RESPONSE TO COMMENTS BY THE REVIEWERS

This document provides detailed comments to the reviewers.

The original letter of the reviewer is always shown at the front of the line (set in a different, serif font: Times New Roman). Our response is set in a sans serif font and indented.

REVIEWER RESPONSES:

Reviewer #1: In revising the manuscript the authors have altered their text thoroughly according to specific suggestions of the other four reviewers. In responding to the five major criticisms formulated in my initial review, they invested a lot of text to justify their approach, unfortunately their action not leading to any changes related to my feedback to the revised text. Specifically, the authors formulate the challenge to introduce any changes related to my feedback, because the other reviewers did not state similar criticisms. I share this feeling to some basic extent as an author of scientific publications myself.

However, given the way, how I formulated my initial criticisms, i.e. scientifically based on the state of current knowledge in the field of motor control, I have some trouble to follow the arguments of the authors towards my initial concerns in the light of serving some majority vote. I have severe trouble to capture this notion in light of the task of scientists to move a specific field forward in understanding by means of scientific sound publications.

Given this situation I see it as task of the receiving editor to judge on the relevance of the reviewers comments to be relevant for a decision.

> We are sorry if the reviewer comes up with the impression that we didn't follow up on his suggestions. We tried to take the comments of all reviewers into account – but having to deal with five reviews that point out different points and in some cases ask for contradicting things to do, leaves us with having to decide how to respond to all the remarks. This holds true as well for the remarks by this reviewer – but it has to be noted (and he explicitly states this) that he offers only some major criticisms. We will briefly reiterate his original list in order to point out that this made it in many instances difficult to really find which issues he wanted to be addressed by us and that in other cases we did actually address these:
>
> First, he points out *"issues are discussed, which have nothing directly to do with the present study"* – it is hard to know which issues he meant by this statement. He explicitly mentions two parts that requires our attention: First, searching movements – we have added at the end of this document a page of the provided automatically generated diff document between initial submission and first revision which shows that we considerably shortened this paragraph. Nonetheless, searching movement is crucial for walking on irregular substrate and in the Discussion we argue which elements of our network might contribute to this behavior. Second, for the ring network we disagree with his assessment that this has *"nothing directly to do with the present study"* as this is a central part of the controller. Furthermore, he asks to split the article in two – as expressed in our response letter we are aware of this option, but

we tried to explain why we think it is important and a benefit to look at a model not on singular experiments, but in the context of a broad range of experiments and he accepted this in his second review round (see below).

His second issue connected to one of his particular interests: he is more interested in details on a muscle model. This is not our focus. Instead we concentrate on relevant properties of embodiment ("loop through the world", the exploitation of which allows for simplification of the neuronal structure), and both is physically not possible at the same time as follows from results referred to by the first Reviewer (v. Twickel et al 2019 and see below). We appreciate that for the revision he provides detailed notes on this point as both approaches complement each other: we therefore added a paragraph on muscle models that introduced all his references and mentions his work, but we think as well (as explicitly stated by this reviewer, too) that a model can focus on different levels.

His third point appeared to be the crucial point and the one that is sparking disagreement: the way we position our paper as an alternative hypothesis for behavioral and neurophysiological results that are often claimed to necessarily require CPGs. We tried to be careful in the way we phrased this. In response to this reviewer (and some minor instances pointed out by other reviewers where we could clarify this) we changed the text accordingly. As a single example (of multiple instances), we added in the first revision already a further sentence at the end of the introduction: *"Note that this is not meant to neglect the general existence of slow CPGs to control rhythmic behavior. We only ask whether such CPGs are necessary components for slow walking."*

His fourth raised point addressed the question of experiments: *"Except for one "experiment" (Figure 5) the authors only provide exemplary figures of results of the simulation and practically no quantitative data."* (Note that the quotation marks in the above quote are taken from the original review – somehow the reviewer found it necessary to not only question the large number of experiments, but even for the one which he is acknowledging he felt the need to diminish our work.). We object to this assessment of our study – none of the other reviewers seem to share this view as well. To the contrary, for example one explicitly states the wide range of experiments or pointing out that this not only covers behavioral but neurophysiological data as well. We don't see how dismissing simulation work in this way could or should be reflected in the manuscript (in particular as this is a journal on Computational Biology). The reviewer is claiming that there is *"practically no quantitative data".* We strongly object to this suggestion: we show a wide range of quantitative results. Furthermore, the whole model is made available online and can be tested and scrutinized in detail. This is the current standard for dealing with computational models. It is widely accepted in the field that it is a much better way to publish a model than to publish only data produced by the model (the Transparency and Openness Promotion standard has been developed and widely acknowledged by over 1000 journals, including PLOS Computational Biology – it differentiates the handling of data and models; while PLOS is currently encouraging publishing of models, we went further ahead and adhered to the TOP standard on the highest level possible for us as authors – for more details see reply to editors).

In the end he explicitly mentions three further instances and adds questions which should be addressed: forward walking has been explained now in much more detail; for backwards walking we addressed in the review his question on why we chose only these particular rules; and, last, his question on activation of load sensors was already answered in the Method section.

To conclude: we not only tried to provide detailed reasoning and comments to the reviewer already in the first revision, but hope that the above list shows that we made considerable changes to the document that are in line with the reviewer's suggestions (and have been explicitly marked up in a provided diff-document).

The first reviewer furthermore somehow now addresses the work of the other reviewers and calls the editor to the task. We agree that such balancing is the task of the editors. But while this reviewer is claiming that he is pointing out important issues that were not mentioned by the other reviewers, he completely fails to acknowledge anything the other reviewers contributed. First, multiple of the other reviewers provided very long and detailed reviews aiming at improving the manuscript. Second, some of the issues mentioned by this reviewer were explicitly addressed by other reviewers, but with completely different conclusions – for example, on the question of the range of experiments, the other reviewers clearly distinguished multiple experiments, one explicitly pointing out the *"wide range"* of simulations; or as a second example and with respect to the main talking point of this reviewer on the role of central pattern generation, the contribution and the specific goal of this study is explicitly mentioned by multiple of the other reviewers (e.g. reviewer 3: *"The manuscript discusses a long-standing issue in the control of legged locomotion: are there central neuronal circuits that produce and drive rhythmic leg movements or are there simpler solutions? … network does not feature any rhythmic central components, but instead relies mostly on sensory input … By testing this network in a variety of conditions that have been explored both behaviorally and neurophysiologically they demonstrate that solutions to all challenges emerge from their network architecture… This manuscript thus provides a compelling alternative to current views on how rhythmic leg movements are produced")*.

In the following, the review will give the relevant parts of the authors response marked by ">" with my comments stated below with "Rev:" preceding.

Response to my initial point 1: > *Point 1: "We are aware that the manuscript is quite long in its current form. We tried to shorten (following explicit suggestions from reviewer 3) where possible. But, from our point of ………"*

Rev: Still I am not comfortable with the concept of the authors in presenting their study, but in case the journal format of PLOS Computational Biology includes studies of that kind, I agree.

From our understanding PLOS Computational Biology is open for such a submission and the checks through PLOS Computation Biology did not see any issue here *("Manuscripts can be any length.")*.

Response to my initial point 2: > *Point 2: "Reviewer 1 criticizes that our dynamic simulation leaves the "most important property unrepresented", namely the relevance of muscle properties. Reviewer 1 is pointing out that there is a large number of nice simulation studies that focus on this aspect and can account well for these properties. We didn't intend to downplay these important contributions and are fully aware of the relevance of muscles and their properties (we have therefore quoted the modeling approaches relevant for us in our Methods section, now lines 1477 – 1504). But we are simply focusing on a different level of description in our approach………."*

Rev: I agree fully with the notion of the authors to restrict their modelling work to a specific level of processing in motor control for locomotion, here the presumed neural level. However, given the fact that the authors aim to place their study within the state-of-the-art knowledge on one thoroughly studied model system, i.e. the stick insect, serving for most of the used results for modelling, it is of upmost scientific importance that they place their study in the hallmark of the currently available knowledge available on this animal. In doing so, they need to include reference to knowledge that was not included. Reference has therefore to be given to relevant publications on recent neuro-mechanical properties of the stick insect motor system, e.g. published by Blümel et al. 2012 (three publications), Hooper et al. 2006, 2007, 2009, and von Twickel et al. 2019.

> We think the reviewer raises a valid issue – therefore, we added a section in the Method parts discussing muscle models in insects and the important work of the reviewer in this area (all line numbers for the changes in the second revision are wrt. the provided diff-document: line 1087–1099). Importantly, we see the mentioned papers as complementary to our work. Our focus is not on a detailed muscle model as stated in our answer and as well as in the manuscript (following reviewers' comments). The reviewer mentions here mostly work from a single group – we find these sufficient as a model of muscles is not the focus of our paper.
>
> We agree with the reviewer that one has to focus on a specific level or specific part – and from our point of view these papers show this as well: They provide nice contributions, e.g. the von Twickel paper (2019, Current Biology) provides a connection of experiments on the measured torques in stick insect and a model. But, importantly, these measurements and the model is formulated for swing movements only – there is no stance movement simulated and as a consequence no interaction between legs or body movement included. This is an excellent work and exactly characterizes the reason why we did not focus on detailed simulation of muscles. v. Twickel et al's detailed study refers to the basic difference between "small limbs" and "large limbs". It is physically not possible to combine tests of a detailed muscle model required for driving a stick insect "small limb" leg with a "large limb" robot (required for including the loop through the world). We decided for the latter case, as this appears to us more relevant to understand control of complex behavior. Important for our work is that the leg trajectories approximate those observed in the insect. This appears even more sensible, as in our simulations gamma joint angle uses a range of about 70° and 80° for stance and about 80° and 100° for swing, each operating in a nearly linear range as shown by v. Twickel et al (2019). Furthermore, we rely on three quite different joints, not just the gamma joint. As stated: this discussion has been added to the Method section.

Response to my initial point 3: > *Point 3:" Another concern addresses the role of network components of the simulation (Introduction Responses to Reviewers – Decentralized control*

of insect walking 5 and Figure2): In the introduction the authors try hard to relativize the functional significance of the ubiquitous finding that central neural circuits generating locomotor activity in many (if not all) studied rhythmic locomotor forms, i.e. swimming, flying, walking, and crawling, contain elements or networks that can generate rhythmic activity without any phasic input, so called CPGs.

In short, Reviewer 1 states that CPGs necessarily contribute to walking. This is exactly the statement that we are challenging (see below). We tried to clarify this in the introduction………………"

Rev: This statement is wrong. My initial review did not state, what the authors are quoting.

CPGs are neural networks in the central nervous system, which can generate a rhythmic activity without any phasic external input, shown in some systems by pharmacological activation.

The authors incorrectly cite the locust flight system as an example for a motor system driven by CPG activity: in contrast to the authors' statement, the locust flight system was the first, for which Pearson and colleagues have shown that it is sensory feedback (e.g. Pearson & Wolf, 1988; Pearson & Ramirez, 1990 and further articles), which affects and sculpts the activity of a centrally generated motor activity by impinging on the network in a way that the CPG only serves in providing the network topology for the externally induced information to act on.

We agree with the characterization of CPGs in the first paragraph mentioned by the reviewer and think that it is in line with how we use the concept in our manuscript. But it is our impression that the findings of central patterns is often simplified to the too narrow view that there is no alternative explanation and that rhythmic activity only stems from CPGs. Our proposed model is challenging this as the only and a necessary condition for any walking.

Again, we didn't want to add to the confusion and are sorry, if the reviewer has the impression that his opinion is wrongly characterized. We think it is important to point out that we tried to further clarify this in the manuscript: first, other reviewers agreed that this is a common misunderstanding and pointed out some instances in which we could clarify this further (importantly, they were very clear on what our goal and position was, they only found that in some instances this was only implicit and should be made explicit). We followed this advice which solved this issue for the other reviewers. Furthermore, we already added an explicit statement in the last revision to avoid this misunderstanding at the end of the Introduction: *"Note that this is not meant to neglect the general existence of slow CPGs to control rhythmic behavior. We only ask whether such CPGs are necessary components for slow walking."*

The reviewer further points out that he disagrees with our view on the locus flight system in our response to him. We are fully aware of the contribution of Pearson and colleagues, but we are not sure if we completely understand the reviewer's reasoning. As we don't argue this in the manuscript in any detail, we simply deleted this phrase to avoid misunderstanding.

The same holds for results from previous studies on walking in insects, in which sensory feedback from the legs was shown to use central neural network topology in contributing to the stepping motor output (e.g Akay et al. 2007; Bucher et al. 2003; Hess & Büschges, 1999).

We are grateful for referring to these three papers as they allow us for clarification. We will not deal with the paper Bucher et al. as it deals with control of the stomatogastric system in lobster, not walking. More interesting are the two other papers (Hess and Büschges, 1999 and Akay et al., 2007).

We will focus on the latter as it is the more detailed and the more recent one. Basically, these papers show that sensory feedback is important for walking.

From our point of view, a detailed interpretation of the results appears difficult because several aspects are not clearly specified. Leg movement on slippery ground, elicited by mechanical stimulation of the body, is interpreted as walking. However, this might also be interpreted as searching movements as the mechanic interaction with the environment completely differs from walking. Furthermore, the leg fixed to a lever to admit load stimuli is considered as in prolonged stance, but it might as well be represented as a prolonged swing. Therefore, these results allow for different interpretations.

Specifically, Akay et al. (2007) show that during active walking stimulating specific campaniform sensilla (CS) switches from protractor activation to retractor activation, which might be interpreted as to switch from swing to stance. As such, this is not unexpected as it is known that sensory input influences the walking rhythm (possibly apart from very fast walking). Interestingly, the authors show that such an influence can also be observed when the insect is deafferented (except the CS input) and treated with pilocarpine. They also show that the CS influence does not influence the motor neurons directly, i.e. beyond the system that is responsible for the oscillations elicited by pilocarpine. As a next step the authors assume that the networks showing artificially elicited oscillations are exactly those structures that are responsible for controlling the walking rhythm, which is based on the scientifically sensible assumption of assuming a minimal structure (Ockham's razor). From our point of view, this introduces the problem that they take this as a starting point and a necessary assumption without considering other possibilities or explanations. Here, we want to point out the difference to our approach: We don't take this as given and the cause of any rhythmic locomotion. Instead, we assume that control of walking "rhythm" is located at a higher level, the network shown in light grey in Fig 2. That means we have two networks, one, the PMN net, does oscillate only when treated with pilocarpine, and the light grey net that allows for quasi-rhythmic motor output, but not as a central pattern oscillator, rather — as it has sometimes been called —, a peripheral oscillator (i.e. based on sensory input).

This means that a structure like neuroWalknet is also able to describe results as provided by Akay et al (2007). So, we think that this is an important paper that allows us to point out the different views of our approach and that of authors from the Büschges' lab. Concerning walking, as a conclusion we argue that, instead of using three CPGs per leg, our approach using no CPGs and only one bistable monopol per leg

is (i) a possible alternative view which has (ii) advantages on the functional level as characterized in our paper. This is now added to the Discussion (line 849–865).

Response to my initial point 4: > *Point 4: "Reviewer 1 states that we do not present quantitative data except for Fig 5. We disagree with this view. From our point of view, the figures show quantitative data, as does figure 5 (in particular, figure 5 represents an abstracted version of figure 3 and a summary of the results of that figure)………."*

Rev: The reviewer still emphasizes that figures 3, 4, 6, 7, 8 and 9 in the revised version of the manuscript give examples of th (from here on the rest of the text is missing, and, although we asked for it now multiple times, we did not receive a complete version of this text)

As mentioned above: We disagree with the view from the initial review by reviewer 1. We do think that simulations provide quantitative results and that the proper way of dissemination of models is to make the model itself available (as argued above and accepted plus encouraged by PLOS Computational Biology). We stand by our original results and the provided model – to which none of the other reviewers object and which adheres to the guidelines of this journal.

Reviewer one further answered on the question: Have all data underlying the figures and results presented in the manuscript been provided? – Rev 1: None

As mentioned: the model is completely open-sourced and everything is made openly available. In the first review round this was as well acknowledged by reviewer one who now (without giving reason) selected "None".

Unfortunately, the review was not submitted in full — and even after asking for the full review multiple times, still only the partial review was made available to us. From our point of view, the mentioned issues appear solvable (first, the reviewer appears to be OK with the format of the paper when it adheres to PLOS requirements; second, we added all his proposed references and discussed our choice of muscle model; for the third point we tried to further clarify and added his raised issue as well as the pointed out references).

Reviewer #2: The authors have addressed all my comments and suggestions. I strongly recommend that this important paper be published.

> We want to thank this reviewer again for his thoughtful comments in the first review round and his suggestions. From our point of view this helped a lot to improve our paper.

Reviewer #3: The authors have responded to all my comments and edited the manuscript appropriately. I have only a few minor suggestions left, none of which should delay the publication of the paper. I use the line numbers from the annotated pdf.

We also want to thank the reviewer for his work – he provided detailed comments, helping to further focus the original message of the article.

He is mentioning some minor remarks for which he explicitly points out that these should not hinder publication. We found these suggestions reasonable and easy to realize.

490 – 530: Maybe a bit too wordy. The sentences could be more concise.

Shortened

529 "Tests with varying parameters showed that application of different local velocities was indeed necessary to elicit this kind of curve walking. Changing only the AEP of front legs was not sufficient". I am unclear about how necessity and sufficiency were tested here.

Changed and shortened

822. What is meant by 'there are no experimental results concerning I1'? This statement is either too broad or the context is not clear.

We deleted this phrase as it is not relevant here

890 Change to "does not oscillate EITHER.'

Done

962 and other places. Like one of the other reviewers mentioned, "mv" for velocity appears unusual.

All relevant cases are changed in “velocity neuron set to”

1001: 'as addressed in Results' makes no sense since this is now in the results section

Done

1371: The writing of the new section starting in line 1368 seems a bit clunky and could be shortened. For example "As detailed data are available for one specific case only, we could not aim for a general solution, but, in order to approximate data of Dürr and Ebeling (2005), chose plausible set point values for". Did you mean "As detailed experimental data are only available from Dürr and Ebeling (2005), we chose plausible set point values that approximated..... ?

Changed accordingly

Similarly, in line 1380 "As there is no detailed information given concerning the outer leg" It would be better to make clear that Dürr and Ebeling do not give this information (vs you not having that information) and reformulate the sentence to make it simpler.

Shortened

1385 "Again, as only this data set is available, we did not attempt to find a general solution for all possible turning radii, but focused on this special case. " Sounds very repetitive (maybe because you were trying to satisfy another reviewer).

Shortened

Reviewer #4: I am satisfied with the changes the authors made to the manuscript and recommend acceptance.

Thank you for your review and assessment of our manuscript during both review rounds.

Reviewer #5: The authors addressed all my comments satisfactorily and revised the manuscript accordingly. I have no further comments.

Thank you for your review and assessment of our manuscript during both review rounds. From our point of view, the suggestions helped avoid misunderstandings and helped to clarify the manuscript. In the Discussion we added a brief reference to a recent paper that further clarifies the point.

DATA AVAILABILITY STATEMENT:

Have all data underlying the figures and results presented in the manuscript been provided?
 Large-scale datasets should be made available via a public repository as described in the <i>PLOS Computational Biology</i> data availability policy, and numerical data that underlies graphs or summary statistics should be provided in spreadsheet form as supporting information.

Reviewer #1: None

Reviewer #2: Yes

Reviewer #3: Yes

Reviewer #4: Yes

Reviewer #5: Yes

As mentioned: the model is completely open-sourced and everything is made openly available. In the first review round this was as well acknowledged by reviewer one who now somehow (without giving reason) selected “None”. The editors appear to follow this assessment and didn’t take into account that we mention the availability of the model in the paper and supplement.

CHANGES IN ORIGINAL DOCUMENT ON SEARCHING MOVEMENTS

Neural representation of Searching Movements as part of an Adaptive Swing Movement: In stick insects, [I59]searching movements of a leg are observed if the leg at the end of the swing movement is not stopped by ground contact (Dürr 2001). Frequencies of searching movements are about 3 Hz (and about 10 Hz in *Drosophila,* Berendes et al. 2016). In both cases, these values correspond to that of the uppermost leg frequency during fast walking. ~~Starting from our initial assumption (see Introduction), one could~~One may therefore ask if such searching movements represent a distinct behavior which would be addressed separately on the level of motor selection, or if searching movements just simply are part of an adaptive swing behavior. In an excellent study concerning the control of searching movements, Berg et al. (2015) investigated the contribution of non-spiking inhibitory interneurons during stance and during swing as well as during performance of searching movements in a restrained middle leg and found two antagonistically active groups. One group is hyperpolarized during flexor activity, while neurons of the other group are depolarized during flexor activity. Two neurons, I1 and I4, each belonging to one of either group, have been studied in more detail. I1 belongs to the former group and is active during stance, I4 belongs to the latter one and is active during swing movement and during searching movements in *Carausius morosus*. Berg et al. (2015) could show that swing and search is stopped either by foot contact or by hyperpolarization of I4. Artificial neurons of this type are found in neuroWalknet, too, whereby the lower light grey unit in Fig 2 may correspond to a neuron like I4 and the upper light grey unit may correspond to a neuron like I1 (~~no information is given as to~~ there are no experimental results concerning [I60]the effect of neuron I1). If this interpretation is correct, an additional neuronal unit representing a searching movement would not be necessary, contrary to what has been postulated by Berg et al. (2015) and tested in a simulation by Szczecinski ~~et al.~~and Quinn (2017). Therefore, swing and search behavior may result as an emergent property from the network already required for basic walking. ~~In this view~~Consequently[I61], the oscillations observed during searching result from the properties of negative feedback loops controlling the joints during swing as given in neuroWalknet (~~not simulated) and as has already been proposed in a simulation by~~see also Dürr (2001).

~~If at the end of swing, during touch down, the tarsus does not find efficient adhesion to the substrate, the leg may react by lifting the leg up and down similar to that of a searching movement. This behavior corresponds to what has been described as "short steps" (Theunissen and Dürr 2013; Bläsing and Cruse 2004) and might be termed short step A (for anterior) in contrast to short step P (for posterior, see Methods and Supplement). As short step A may be caused by the same structure as used for searching, but under specific environmental conditions, we did not simulate this behavior, in contrast to short step P (see Supplement).~~
