# Supplementary material for: Decentralized control of insect walking: A simple neural network explains a wide range of behavioral and neurophysiological results
Source: PLoS Comput Biol. 2020 Apr 27;16(4):e1007804. doi: 10.1371/journal.pcbi.1007804 (PMC7205325; doi:10.1371/journal.pcbi.1007804)
Supplement: Attachment — Submitted filename: 2019_11_04_ReviewerResponses.docx [file pcbi.1007804.s022.docx]

RESPONSE TO COMMENTS – ALL REVIEWERS

First, we want to thank all the reviewers and the editor for the consideration of our manuscript as we are aware that the paper is quite long. From our point of view this is a crucial contribution as we try not to reproduce a single specific experiment or a single specific type of experiment, but instead present an architecture that allows to address quite diverse types of experiments. Overall, it is our impression that most of the reviewers agree with such a view and see this as a contribution in itself. Therefore, we will stick with the general structure of the article.

Further, thank you for all the suggestions – again, as the article is quite long there are many suggestions and we think that many of these helped us to improve the article further and include more connections to related work.

There are some general changes we want to point out:

- we added to the introduction a section discussing the notion of running and try to disambiguate this better.
- For the introduction of a walking system (now line 288 and following): we structured this part and provide more basic information introducing the coordination rules.
- We moved the result section on curve walking from the supplement to the main text (as proposed by reviewer 3, now starts at line 599).
- Furthermore, we tried to shorten parts of the article as pointed out by reviewer 3.

We provide a difference Word document that highlights the changes made. As a remark: Unfortunately, line numbers depend on how the file is processed and the online submission system produces quite different line numbers. Therefore, we added comments in the difference document for many of the reviewers remarks that allow to relate these to your suggestions or questions. In addition, in our responses, we provide the new line numbers for a Word difference document which we uploaded to <https://www.dropbox.com/s/9xk91s6blxwvc2c/Diff_schilling_cruse_neurowalknet_2019.docx?dl=0> and which should provide the correct line numbers. We are sorry for this inconvenience, but the online submission system is unfortunately not producing stable line numbering for the difference document.

In the following, we will address the reviewers’ comments one by one.

REVIEWER 1 RESPONSE:

Reviewer #1: The study of Schilling and Cruse addresses a timely topic in the field of motor control, i.e. how control architectures can contribute to the generation of a highly adaptable walking behavior with respect to the generation of a broad range of stepping velocities. In the course of the study, the authors present a new control architecture, neuroWalknet, extending the well elaborated simulation Walknet. Using this simulator the authors show potentially very interesting data on the generation of interleg coordination patterns changing with walking speed of the simulator and interesting evidence for their controller architecture to generate motor activities similar to those found in deafferented and pharmacologically activated thoracic ganglia of insects. Such study might be interesting for a broad audience of scientists in the field of robotics and motor control.
However, in its present form the manuscript shows shortcomings that need to be dealt with by the authors in order to allow a reader to follow and assess the results. I have therefore only listed my most major criticisms.

1. The manuscript is on the one hand far too extensive, even for an informed and highly interested reader. On the other hand, it still lacks important information for backing the conclusions drawn by the authors – major points 2 - 4 potentially directly arise from this current drawback.

We are aware that the manuscript is quite long in its current form. We tried to shorten (following explicit suggestions from reviewer 3) where possible. But, from our point of view, it is crucial that we present a single system that can deal with a set of different experimental situations and in particular with quite differing experimental paradigms instead of showing this for a single narrowly constructed experimental setup. As argued in the general remarks, we therefore would like to stick to the current format, as this has been valued by the other reviewers.

As the reviewer is pointing out that the article is lacking important information, we want to point out that the whole model is openly available and experiments can be reproduced as well as further experiments run against our model.

We will deal with points 2 – 4 below separately.

The reason for this situation appears to be that the authors not only present data on their three research questions in focus based on new simulation studies, but also present a completely new simulation of the stick insect walking system.

Reviewer 1 reports critically that we treat three research questions plus a completely new simulation. As argued, from our point of view both these aspects are tightly coupled. We deal with a large number of behavioral and neurophysiological results that can be described by one network. A separation between network and results of these biological studies is not sensible.

Furthermore, neither the introduction, nor the discussion is well focused. This is in particular obvious from the discussion, in which also issues are discussed, which have nothing directly to do with the present study (but perhaps with the new simulation), for example, the generation of searching movements (line 665 and following)

From our point of view, the goal of simulation studies of biological systems like ours is twofold: First, it should account for experimental data and should present hypotheses describing as many biological data as possible. Secondly, and this is not addressed in many cases, it should present predictions that could stimulate new experiments. The present study is based on the “new simulation” and we aimed to include both aspects: first, properties of the simulation system should directly be inspired and explain biological results. And secondly we aimed in the Discussion for discussing further results and how these relate to our model, how they could be explained by our model and proposed how this could be verified. From our point of view, these two aspects cannot (or should not) be separated.

In particular, the attempt to find an explanation for the control of searching movements, an important aspect of walking in difficult environment, concerns a central point of our work. Therefore, by introducing neuroWalknet, we present a very simple explanation. Importantly, there are competing models (e.g. proposed by Berg E, Hooper, Schmidt, Büschges, 2015; or a simulation by Szczecinsky and Quinn,2017) which appear more complex and rely on more basic assumptions. Thus, our attempt has “directly to do with the present study” aiming to describe as many biological results as possible.

and the existence of the ring networks in the simulation (line 969 and following).

(line 969 mentioned by the reviewer is probably a typo; he probably refers to line 696, now line 1034)

The ring networks are central elements of the leg controller. Therefore, we are not sure how these potentially can “have nothing directly to do with the present study”.

I strongly suggest that the authors consider dividing this manuscript at least in two, one presenting the new simulation together with the necessary detailed presentation of its composition and its testing and a second dealing with the three present questions in focus.

Reviewer 1 suggests splitting the paper “in at least two”. As argued in the general remarks for the reviewers, we don’t aim to find models in single experimental settings, but aim for a model that spans a whole continuum of experiments and in this case even spanning experiment paradigms. In our case, simulation for interpretation of many biological results (we want to add that this includes much more than three experiments) would make such a split even more difficult for the reader. Therefore, we would like to keep the general structure as it is. This view is supported by the four other reviewers who do not make such a proposal and apparently like the actual structure. In particular, Reviewer 3 makes a number of very constructive proposals to improve readability which we very much appreciate and tried to follow them.

2. One concern is directed towards the concept of the simulation (line 271 and following): given today’s knowledge about the relevance of muscle properties for the generation of movements and the existing wealth of literature it appears puzzling that the authors generate a dynamic simulation that essentially leaves this most important output level of movement generation unrepresented. Detailed information on muscle properties is available for the simulated organism, i.e. the stick insect, making it with respect to muscle properties one of the most thoroughly studied insect species in that respect. This shortcoming is even more relevant as the authors argue that for their research concept closing the loop through the environment is crucial and as they aim with their simulation for a holistic description based “on a minimum of a priori assumptions” allowing for a more complete testing of conclusions drawn from experiments (line 286 - 298).

Point 2: Reviewer 1 criticizes that our dynamic simulation leaves the “most important property unrepresented”, namely the relevance of muscle properties.

Reviewer 1 is pointing out that there is a large number of nice simulation studies that focus on this aspect and can account well for these properties. We didn’t intend to downplay these important contributions and are fully aware of the relevance of muscles and their properties (we have therefore quoted the modeling approaches relevant for us in our Methods section, now lines 1477 – 1504). But we are simply focusing on a different level of description in our approach.

From our point of view, simulation in general always requires to focus on certain aspects which leaves out other aspects as well. In particular, for a minimal approach this does not mean that any detail has to be taken into account. Quite in contrast, it should be as simple as possible and only implement those elements that are crucial to describe the goal being addressed (“minimum of a priori assumptions”). Here, we assume that we simply have a different focus compared to the reviewer. We wouldn’t agree that elastic properties represent the “most important property”. While, for example, their elastic properties appear crucial for running, it is our hypothesis (which was shown on a different level of description earlier on multiple times for the original Walknet approach) that sensory feedback contributes to the control of (slow) walking and benefits adaptivity. This requires a full dynamical simulation that provides a loop through the world (Brooks, 1991) for sensory feedback. Such an embodied approach has been shown advantageous as it allows to exploit the interaction with the environment which actually can simplify computation (e.g. our own work on coordination of multiple legs during walking, Schmitz et al. 2008). We want to point out, that, importantly, adding a dynamic simulation (or implementing such a system on a robot) is therefore not only adding another layer of detail to a model, but it is crucial closing the loop of the interaction with the environment. The simulation offers feedback of the environment which is a crucial part of natural behavior that from our point of view should be included in a model.

Integrating detailed muscle properties might be a further step, but is from our point of view beyond the scope of the current article. Currently, (simplified) muscle properties are represented in our simulation (now lines 1482, 1488, 1493) and the robot Hector actually is equipped with such muscle properties including an integral position negative feedback output. We believe that it is not necessary to simulate the muscles in more detail for reaching our current goal (furthermore, as motor output and torque may operate quite independently, see Dallmann et al. 2016, and Supplement for a brief discussion).

3. Another concern addresses the role of network components of the simulation (Introduction and Figure2): In the introduction the authors try hard to relativize the functional significance of the ubiquitous finding that central neural circuits generating locomotor activity in many (if not all) studied rhythmic locomotor forms, i.e. swimming, flying, walking, and crawling, contain elements or networks that can generate rhythmic activity without any phasic input, so-called CPGs.

In short, Reviewer 1 states that CPGs necessarily contribute to walking. This is exactly the statement that we are challenging (see below). We tried to clarify this in the introduction.

One part of their study aims for providing evidence, how their decentralized neural control structure can explain neural oscillations without an explicit CPG network organization involved (at least for low cycle periods). When inspecting the topology of the simulation presented in Figure 2, it becomes immediately clear that each joint control network contains the architecture for a CPG in the circuit connecting the PMNs via mutual inhibition, a circuit scheme ubiquitously found in CPGs and assuring the generation/maintenance of phases of activity (such minimal network will assure a two-phasic activity). The network topology used here represents the ideal form of a so-called half-center CPG. Please comment and provide evidence that the simulation also works, when removing these CPGs.

First, we want to clarify that we do not argue that swimming and flying do not rely on CPGs. Neither do we argue for walking of non-arthropods, which have quite different morphology. We focus on insects (and to some extent on crustaceans).

Second, we want to point out that the purpose of our simulation is exactly to analyze parts of a functioning model or structure and looking at the contributions of different parts in a running system. This allows to go beyond a schematic view on a topology and draw conclusions on the functioning of a system. Our argument that this model does not use an explicit CPG during walking depends on four different points as addressed in Methods (now lines 1908 – 1920). Importantly, the structure mentioned by the reviewer, is not active in normal walking behavior: In particular, it depends on a large enough input to both antagonistic units, termed “endogenous input” by Bässler (1986). In our case these inputs (shown in Fig 2, “pilo”) are set to zero in all simulations apart from those where (simulated) pilocarpine is applied. Therefore, when simulating the intact animal, this network cannot produce central oscillations, and therefore there are no functional CPGs in the intact animal. Rather, there is only antagonistic input that depends on antagonistic sensors.

The input of this network results, depending on type of joint and state (swing – stance), from error signals computed by a negative feedback controller (positive error to the agonist, negative error to the antagonist) or from the ring network that also provides the signal to either the agonist or the antagonist (see Fig. S2A). Therefore cocontraction may only appear during the transitions due to low pass filter properties of the neurons. The function of this network connecting PMNs operates as lateral inhibition to minimize co-contraction.

Only if a crucial change is applied to the circuit – in biological terms, only if legs are deafferented and pilocarpine is given, – a situation quite different from that of an intact insect, the network properties are dramatically changed which then allows for oscillations (now lines 1904 – 1920).

This misunderstanding may be caused by a misleading formulation on line 474, using the term “load sensors”. What we meant is explained in detail in Methods (listed on line 1900 - 1920). To avoid this misunderstanding, we have now reformulated this sentence to:

*“Furthermore, the mode of each leg was set to stance and the inputs to the PMN units, marked by “pilo” in Fig 2 (and Methods) were set to high values, thereby simulating the effect of deafferentation and pilocarpine”.*

Further, Reviewer 1 asked to “provide evidence that the system also works when removing these CPGs”. As already explained above, there are no CPGs (or intrinsic oscillators) in the walking simulation. All walking studies operate without using the claimed CPGs, but use lateral inhibition. Their function is to avoid/minimize cocontraction of antagonists. Properties of CPGs are only occurring in preparations treated with pilocarpine. So, evidence is provided by results shown in Figs 3 – 6.

Our question is not if there are CPGs to be found in an insect neural system, but if the CPGs observed when treated with pilocarpine are responsible for controlling walking movement. This is a quite difficult question when dealing with an animal (see the still very relevant excellent papers by Bässler, 1986, and Pearson, 1987). However, in a simulation study this is possible, because here the situation is very different as the case is clearly defined. It is known if a CPG is implemented and activated and what exactly is its function in the system.

We responded to a related question of Reviewer 5, his/her comment number 4, by:

*“we do not state that these animals do not use CPGs for control of walking. We however state that the currently available data may well be explained without requiring CPGs for the control of hexapod walking. And we furthermore state that even those data, which are often used to prove that CPGs do control insect walking for pentapod, tetrapod and tripod gaits, may be explained by a neuronal structure that is not equipped with CPGs when the insect is intact.”*

Here we want to point to another possible misunderstanding. The networks controlling either the states swing – stance or the states forward – backward (grey units in Fig 2) are no CPGs, too. In these networks the inhibitory channels do not contain phasic properties (HPFs). Both networks are therefore no CPGs, but so called classical bistable monopoles. These monopoles can switch the state only when both inputs (from sensors or via coordination rules in the case of state Swing – Stance) show strong differences. To summarize this point, as accepted by all the four other reviewers, our simulator does not operate with CPGs as long as the (simulated) insect is intact (no pilocarpine).

4. Except for one “experiment” (Figure 5) the authors only provide exemplary figures of results of the simulation and practically no quantitative data. This unfortunately renders their conclusions presently as claims only. This applies for all sections, i.e. “Forward walking – emerging gaits”, “Backward walking”, “Analysis of neural activity – the possible function of Intrinsic Oscillation”.

Reviewer 1 states that we do not present quantitative data except for Fig 5. We disagree with this view. From our point of view, the figures show quantitative data, as does figure 5 (in particular, figure 5 represents an abstracted version of figure 3 and a summary of the results of that figure).

The reviewer’s comment appears also at odds with the other reviewers who did accept simulations as experiments. Even more, for the revised version, reviewer 3 pointed at data for curve walking which we only provided in the supplement. Initially, we were reluctant to put this in the main text as this only shows a single trial of curve walking because there is basically only detailed data on a single run in the literature. But, this has been moved following the suggestion of reviewer three to the results section (now Fig. 4).

As one further point: we want to point out that the model and simulation environment is made open access. This follow the current trend in Computer Science and related fields where open models are becoming slowly a gold standard (or even a requirement for publication). Open access of model and environments allows for full reproducibility as well as testing the model in novel experimental conditions or in varied conditions. We are not aware of many models in this area (if at all) which have been made openly available and allow for further experimentation. Therefore, we strongly disagree with the notion of only providing anecdotal data, as it is easily possible to reproduce our experiments, for example, showing for any intermediate velocities (besides all the values that we already covered in the article) the emergence of walking behavior and patterns.

We first respond to this statement concerning the sections Forward Walking and Backward Walking, and will later deal with section “Analysis of Neural Activity”

Here are some selected examples out of many more instances of the study for relevant issues to be addressed with quantitative data.
Line 368 - 431: At the moment, for example, it is not clear, how the authors can come to the conclusion that their simulation is generating a continuum of gaits with increasing voltage.

This follows from the fact that these patterns result from changing stance duration, which directly depends on walking velocity (given as voltage, any value between 8 mV and 50 mV is possible), see now lines 414 and 1500. We show the emerging stable patterns for many different example voltage inputs. The inputs were varied systematically (starting from the lowest sensible value to the highest sensible value – these two conditions were found using inspection of the resulting walking behavior – and covering the range in between systematically) and lead to a large number of runs. Furthermore, as mentioned above, as the simulator and model is open sourced, this can be reproduced and further tested for different velocities (not shown in the article).

Also, the fact needs explanation that the maximal stepping frequency for a single leg apparently does not exceed 0.2 Hz (Figure 3A), which is even by far slower than a stick insect walks.

Stepping frequency: As mentioned in Methods, now line 1500, and now line 589, Results, the simulation shows a 1:10 relation concerning time scale processes between robot and stick insect. A 1:1 relation is physically not (yet) possible.

“by far slower than the stick insect walker”: real time is not a necessary requirement for a simulation approach, and appears often not sensible (e.g. consider any predictive model for climate change which should not run in real time).

Line 432 - 438: How does backward walking result from activating rule 1 and 2i, what other solutions were tested and what is the experimental evidence to select these two rules?

This has been explained in the article: How does backward walking result from activating rule 1 and 2i? First, it basically results from switching the motivation units “forward” to “backward”. Second, during backward walking, rules 1 and 2i function in the same way as during forward walking (as described now in lines 1846 – 1849). Third, there are not enough biological data to provide solid experimental evidence. Therefore, we tested different solutions to find a minimum number of rules. Using only rule 1 was not sufficient. Furthermore, we had to add a specific rule for backward (rule 2c backward, Methods, now lines 1846 - 1849), also mentioned in Discussion, now lines 1278 – 1291.

Our simulation represents a hypothesis to stimulate biologists to support or falsify this hypothesis, an important function of simulation studies. This result, for example, might be relevant for a recent study of Szczecinski, Bockemühl, Chockley, Büschges (2018, Jorunal of Exp. Biol. 221, Static stability predicts the continuum of interleg coordination patterns in Drosophila).

Line 472 - 506: What values were tested for the load sensors and what was the resulting activity of the premotor neurons? How can be excluded that the results are a consequence of the built-in CPGs (see above)?

This was explained in detail in Methods (now lines 1900 – 1920 and in particular in lines 1908 – 1920) including the strength of input used to represent the pilocarpine effect. In short, CPG properties are only given when pilocarpine conditions are provided. In intact insects (no pilocarpine) there are no CPGs, but lateral inhibition without bilateral endogenous input. We rephrased the sentence now in line 718 to avoid a possible misunderstanding (see above).

In this section three quite different experimental approaches are considered that are characterized by showing specific properties, as are in-phase coupling, anti-phase coupling and bimodal coupling. These are qualitative properties observed in insects, which we tried to simulate and this was quite successful. The interleg in-phase coupling found by Knebel et al (and earlier by Büschges et al. 1995, which also includes loss of intraleg coupling found by the latter authors) is an important result, even more as it can now be explained by other known mechanisms (“rule 5”).

Overall, we tried to follow Reviewer 1’s general suggestion and be more explicit on the scope of our study. We further tried to relate this better to his view. We are not really sure what the reviewer means by stressing that we only provide “claims” and is not acknowledging most of our data as quantitative. In part, we agree if by claims it is meant that our model suggests hypotheses for further experimental validation. On the other hand, from our point of view simulation approaches provide a valid experimentation method which provides quantitative data. Even more, as we are following the current trend on making our model and environment openly accessible, we want to stress that it is possible to fully reproduce our experiments and extend this to more variations or even more novel experimental paradigms.

REVIEWER 2 RESPONSE:

Reviewer #2: This excellent paper presents a sensory based controller for insect walking that does not contain a central pattern generator. This controller utilizes information from sense organs to produce walking patterns and is capable of generating a wide variety of gaits, similar to those seen in insects. Furthermore, the controller effectively resists perturbations, is capable of rapid stops, changes in velocity and can walk both forward and backward.

The manuscript is remarkable for the clarity of the writing and logical expositions. This paper will be of considerable value to the readers of PLOS, including neurobiologists, engineers and roboticists. My only comments are the addition of some references and minor changes that I believe would benefit the reader.

Thanks a lot for your positive remarks and for the comments. They are very helpful and the text is adapted correspondingly

Additional Reference

1- Walking/running requires pattern generator - The authors have very clearly and convincingly stated their arguments. However, it is important to remember that CPGs are defined experimentally as elements that produce rhythmic activities in the experimentally-induced absence of sensory feedback. What is being studied only shows the capabilities of the system that remain after injury. It is likely that the identification and characterization of those elements depend upon the experimental procedures producing the isolation. This was very clearly stated by Kier Pearson in a review article years ago and it would be beneficial to the reader to cite this paper (below).

Pearson K.G. (1987) Central pattern generation: a concept under scrutiny. In: McLennan H., Ledsome J.R., McIntosh C.H.S., Jones D.R. (eds) Advances in Physiological Research. Springer, Boston, MA

Thank you very much for your positive reaction in general and the recommendation of this reference. Actually, following also Reviewer 3, we added the references of Pearson (1987) and of Bässler (1986) that appear supportive to our view (now in line 75, 337, 347, 1099). As recommeded by Reviewer 5, we also added Grillner and Wallen 1985.

Minor Corrections

Abstract, beginning line 18 - Redundant use of the words pattern and include; also the term faculty is somewhat unclear. I suggest this would be better as: This includes the ability to produce footfall patterns such as velocity dependent “tripod”, “tetrapod”, and “pentapod” patterns, as well as various stable intermediate gaits as observed in stick insects and in Drosophila.

We followed the reviewers suggestion, now line 18-19

line 40 - Counterproductive - I believe the authors mean 'counterproductive in robotic controllers'. Evolution does not necessarily exclude neuronal elements based on their efficiency or apparent productivity.

We followed the reviewers suggestion and clarified this to: *“neuronal rhythm generators are not required, but may rather be counterproductive as concerns computational efficiency.”*

line 76 - Better as 'In contrast to' rather than 'Different' to.

We followed the reviewers suggestion.

line 145 - It is also possible that, during rapid walking, sensory inputs in one phase could influence the output in the next phase. This question has not been investigated in insects but the authors might consider and mention this possibility. It might also benefit the reader to cite the study of Holtje and Hustert (below) showing that the conduction delays from sensory receptors indicating leg contact/force located on proximal leg segments are very short and could function at higher stepping frequencies.

Höltje M, Hustert R. Rapid mechano-sensory pathways code leg impact and
elicit very rapid reflexes in insects. J Exp Biol 206: 2713–2724, 2003

Thank you for the comments, we included the study of Höltje and Hustert and expanded the explanation (including the note of the reviewer, now lines 160 – 169 ).

REVIEWER 3 RESPONSE:

Reviewer #3: This manuscript discusses a long-standing issue in the control of legged locomotion: are there central neuronal circuits that produce and drive rhythmic leg movements or are there simpler solutions? In their studies, Schilling and Kruse provide a network of neurons that is based on behavioral data from insect locomotion and controls a simulated six-legged robot at varies walking speeds. This network does not feature any rhythmic central components, but instead relies mostly on sensory input and coordinating influences between the different legs. By testing this network in a variety of conditions that have been explored both behaviorally and neurophysiologically they demonstrate that solutions to all challenges emerge from their network architecture, and that these solutions correspond well to previously published locomotion findings. In addition, emerging from the network responses, similarities to known neuronal structures in insects and other legged animals become obvious.

This manuscript thus provides a compelling alternative to current views on how rhythmic leg movements are produced. It is very well written, albeit somewhat long and redundant. I have no major comments on the scientific conclusions, but several that pertain to the order and logic of the arguments presented. In addition, I have quite a few suggestions that may help to improve the reader's understanding of the text (since this is a very long manuscript).


Redundancies and logic/order:
1. Lines 134 and 157: the continuum of stepping patterns / the continuous transitions into other patters. I think this needs to be elaborated more since this is a concept that is later tested explicitly in the model, but it is not introduced well. How are these transitions continuous? Do patterns flip forth and back between different gaits, for example?

Yes, we agree “continuous transitions” reads strange. This has now been reformulated and placed in better context (now in lines 170 – 246)

2. Lines 176 - 179: switch the order and number them (1 = how to couple, 2 = how to control individual legs). This then fits the order of the next paragraphs, which also should be numbered.

Done, now lines 288 – 334

3. Lines 225 - 233: This paragraph seems partly unclear. Is there a way of improving the description of the difference in phasing?

We refer to contralateral coupling, is now specified, now lines 350 – 368

4. Line 250. Delete this reason, it is not necessary and diminishes your arguments. Similarly, the mentioning of the reasons not to address the Manoonpong study sidetracks and distracts. Also, this helps shortening the introduction.

Done.

5. One thing missing though is the coordinating influences that are later mentioned in the methods section. Since the 5 influences are a substantial and necessary part to understanding the approach, they should be defined in the introduction, and not be hidden in the methods. This is particularly important for readers outside of the stick insect world that are not familiar with walknet or the original Cruse publications.

Done, now on lines 295 – 303.

6. Fig. 5: There are several dashed lines. Mention which ones are which.

Done (in legend).

7. Curve walking, line 422: This paragraph needs to be extended. The purpose of the curve walking test is not clear. Most of the pertaining explanations are found in the supplement. However, the purpose of a supplement is not to provide an additional results part, but instead to supplement a fully understandable and complete dataset that is supplied in the results section of the manuscript. The supplement should provide additional information, not the actual main data for the results.

We first hesitated to present the results in the main text, as it is only a proof of concept approach. But we like very much to follow the recommendation of reviewer 3.

Now negotiating curves appears in Results, line 599 – 654.

8. Backwards walking: the results are essentially absent for backwards walking (except for pointing at the figure). The results need to be elaborated in the text.

Yes, we agree, this was too short. Now with more details on lines 670 – 683

9. Redundancy: Lines 445ff: most of this has been stated before and can be cut. The next page (lines 452 - 475 could be shortened substantially. Is it really necessary to provide details of the previous publications?

As these data are the basis of our simulations we tried to find a compromise between shortening the text and still provide information relevant for understanding our approach. Now lines 699 - 715

10. Lines 544: it is unclear whether this refers to your study or previous experiments. Later in this paragraph: why was the slipping legs chosen as approach?

In the simulation, the robot walks on flat ground. The fixed legs are slipping on the ground. In the biological experiment with stick insects these legs were placed on a platform fixed within the body coordinate system. This is now better explained on lines 818 – 833.

11. Line 631. No this is not the conclusion from these arguments. Would the conclusion not be that the new controller corresponds well to established behavioral data?

Thank you, changed: lines 933 – 936.

12. Line 640, first part: I do not understand what that means. Next sentence: which levels? Next sentence: can be deleted (shortened). Next para: line 643: delete (repetitive).

Sorry for these strange formulations. Now changed and shortened: lines 940 – 943

13. Lines 653 - 660, then 661 - 664: I suggest to reverse the order in these paragraphs (experimental findings first, then model) to match with the subsequent paragraph and to make logic clear.

All done, now lines 979 – 986

14. Somewhere in the discussion I think the old Baessler paper Bässler, U. (1986). On the definition of central pattern generator and its sensory control. Biological Cybernetics, 54(1), 65-69 should be mentioned. It provides a nice discussion about CPG vs sensory control.

Thank you very much. Reviewer 2 pointed to Pearson (1987). We now quote both at several positions.

15. Lines 689: the purpose of this paragraph is unclear.

Now reformulated and moved to Methods, lines 1623 – 1636

16. Line 804: please add what the conclusion is "suggesting that...."

Now line 1251 – 1252.

17. Line 857. This paragraph is largely redundant to other parts of the manuscript. It can be shortened substantially while still providing the conclusion.

Done, now lines 1331 – 1338

18. Line 896, conclusions. Again, too much redundancies and repetitions. I understand you want to make sure the main points are understood but at this point of the manuscript it is unnecessary to repeat the arguments for your conclusions. Just providing conclusions is fine.

Now shortened and focused, lines 1384 following

19. Line 999 ff: "piece-wise linear with a threshold of 0". It is unclear what that means and why this was done given that these are HH models. Similarly, how can a HH model work with no leak? Later: time constants do not rely on leak settings and leak does not have a time constant. This section needs to be improved.

Apparently, it was misleading to use the term HH equation. We now made it clearer that we use a strongly simplified version and hopefully formulated this in a way being short, but understandable. Unfortunately, we originally increased trouble by using “leak”, where we meant time constant and actually provided as numbers 1/time_constant. Sorry for this confusion. This has now been corrected: lines 1506 – 1535

Other comments:
1. Line 52: replace "a" with "some"

Done, now line 63.

2. Line 69: there are quite a few rhythmic biological movements that are driven by well characterized CPGs (with spontaneously active endogenous bursters) that are slow. Those may not be involved in locomotion, but nevertheless drive slow rhythmic movements and should thus be considered in the text here. Invertebrate heartbeat or digestion would be examples.

We refer to this aspect now on line 449 – 451

3. Line 74: Water and air can be quite turbulent and 'unexpected'. It is not clear how that contrasts to walking. Please elaborate in the text.

We reformulated this to make the contrast more obvious: lines 85 – 87

4. Line 77: did you mean "any joints in a single appendage"?

Now explained later (“see below”), line 88

5. Line 107: "tarsi during climbing"?

Done, now on line 130

6. Line 121: "leads to" or "requires" (or both)?

“leads to” on line 150

7. Line 124: replace "and" with "with"

Done.

8. Line 147: In the end you are discussing faster speeds as well so this limitation can be deleted from the text.

Deleted.

9. Line 169: replace "any" with "every"

Done, lines 203

10. Line 184: "which is based on ... ... controllers and coordination influences that mainly regulate..."

Done, line 293 – 294

11. Line 210: delete "not considered in walknet"

Done.

12. Line 247: No, this paragraph lays out questions for the coordination of appendages, not for the functional properties of the CPGs.

Thanks, now reformulated, now line 381 – 382

13. Line 266: ", the simulation requires a body..".

Done, now line 413.

14. Line 270. Reference Fig 1B here.

Done, now line 417.

15. Line 289: "concern" no "s"

Done, now line 440.

16. Line 348: the name 'neurowalknet' needs to be introduced earlier (NOT in the methods section).

Done, now first mentioned and introduced in line 168.

17. Line 369: replace "and with" with "but at"

Done, now line 531.

18. Line 382: add "only" after "reached"

Done, now line 550.

19. Line 402: Explain in the text why it is important to note the average.

Explained now on line 572 – 573.

20. Line 418: explain in the text which "effort" is meant.

Explained on line 594 – 596

21. Line 434: in line with your other arguments - why is backwards walking now a different state (and not one that a single controller could achieve)?

Our controller may have different states (e.g. on the leg level: swing – stance). Concerning forward – backward we made a corresponding decision because we are not aware of a continuum between forward (straight or curved) walking and backward walking, at least for stick insects (that seems to be different in some flies which appear to be able to perform non-holonomic walking). Now line 677 – 683.

22. Line 437: Github: May benefit from a better description of the individual movies on the website itself.

We added a short description to the movies (as in the supplement). In the end, the movies shall be hosted on the publication server (now line 699).

23. Line 452: jargon. I believe you meant "deal with THE RESULTS of a study" (not the study itself).

Done, now line 699.

24. Line 472: did you mean "to mimic deafferentation, we cut..."?

Thanks, yes, now line 716.

25. Line 507: "interesting" or "excellent" studies. While I agree that this is true, I think IF you say they are interesting or excellent, you need to mention WHY this is the case, not just that they are. An alternative would be to just delete these words. For line 507: maybe instead just say that your neurowalknet even handles rather artificial situations that animals had been exposed to previously in experimental conditions.

Thanks a lot. We tried to follow your recommendation also at other places, here line 625

26. Line 535: upon? Later: "i.e. only some legs were able .."

Done, now line 813.

27. Line 556: why is it important to note that? Similarly: line 583.

Deleted, (now line 835 and line 883)

28. Line 615. Provide reference for walknet

Done, now line 921.

29. Line 617. Shorten: Delete "as has been shown"

Done, now line 923.

30. Lines 622 - 625: check the sentences there. Why eventually?

Has been reformulated, now line 927 – 928.

31. Line 634: delete "highly influential"

Done, now line 938.

32. Line 651: did you mean "thereby predicting (or providing) ideas as to how..."?

reformulated, now line 978.

33. Lines 669: check for redundancies.

We think this is relevant, so we did not change this sentence

34. Line 682: no information is given... What does that mean?

Reformulated, now line 1207 – 1208.

35. Line 685: Replace "in this" with 'consequently, the ...". Delete the "(not simulated) and... " part of the sentence - it does not any important information.

Changed as proposed, now line 1031 – 1032.

36. Line 721: WTA definition must be here. There is much room in this paragraph to shorten the text.

Thank you very much. We believed that the term WTA structure would provide a simple hint of what is meant. However, we now realize that this term is not general enough to characterize both approaches. Therefore, we replaced WTA structure by “lateral inhibition structure” and used another term (bistable monopole) to clarify the difference.

The changes concern now text from line 1058 to 1101.

37. Line 745: CTr??

Deleted, now line 1103.

38. Line 747 ff: shorten. jargon: "deal with"

text is reformulated from line 1102 to 1113.

39. Line 765: "for slow walking CPGs are not necessary, but instead may cause rather nega..."

Done, now line 1172.

40. Line 776: no results shown. So what then? Why do you mention it?

Deleted, line 1184.

41. Line 779: In this paragraph and the next: Check for redundancies.

shortened and reformulated, lines 1187 – 1252

42. Line 816: "INdependent"

Done, now line 1264.

43. Line 821: I very much appreciate this argument.

Thank you.

44. Line 846: Can you predict the outcome of these experiments then?

We discuss the case (lines 1292 – 1305), but we cannot provide detailed prediction, as we would first need the biological data. We could have introduced plausible, but artificial data, and curve walking could be shown. But this is not what we are interested in.

45. Line 849: does this refer to chaotic systems? If so, mention it and provide reference.

The system is not a chaotic one. At the bifurcation, the system either moves into the center of the current attractor pattern, but may use a different trajectory, or if there are two attractors (see example Fig. 4), it also might find its way to the other attractor pattern. Now explained in lines 1309 – 1311.

46. Line 877: "later" what did you mean with that?

“an evolutionary later state”, now reformulated, line 1347.

47. Line 952: which "technical' reasons?

Now explained on line 1467, 1468.

We want to thank the reviewer again for all the detailed comments and corrections. We appreciate very much the time and effort in this careful reading of our manuscript.

REVIEWER 4 RESPONSE:

Reviewer #4: The manuscript titled “Decentralized control of insect walking – a simple neural network explains a wide range of behavioral and neurophysiological results” by Schilling and Cruse describes a sensor-driven controller, called neuroWalknet, that is composed of artificial neurons, some of which are meant to simulate the hypothesized functional roles of identified cells in insect nervous systems (mainly Drosophia and cricket) that appear to participate in various forms of hexapod locomotion. The authors make clear more than once that these sensor-based control mechanisms are presumed to play a dominant role mainly in slow walking and exploratory behaviors, while central pattern generators are required for faster and more tightly coordinated forms of locomotion. The manuscript is generally very well written, and the results contribute significantly to the understanding of locomotor control in arthropods. I have only a very few minor comments.

Thank you very much for your positive and helpful comments. All comments are appreciated and we tried to adapt the text correspondingly.

Minor comments:

[Introduction, pg. 3, lines 74-76] Change to: Swimming and flying are operating in fairly homogenous, fluid media. Walking, in contrast, has to deal with a more difficult substrate, providing unpredictable… In contrast to swimming and flying, walking is characterized…”

We followed the reviewers suggestion, now lines 85 – 87.

[Introduction, pg. 4, line 87] “…will begin with a behavioral level of analysis, highlighting the fact that walking comprises…”

We followed the reviewers suggestion (now line 109).

[Introduction, pg. 4, lines 92-94] Shouldn’t this section be subtitled “A continuum of locomotor behavior?” Among arthropods, are all forms of locomotion on solid surfaces called “walking”, regardless of varying speed and differing gaits? Is “running” really classified as a very fast form of “walking?” I suggest that lines 93-94 should read something like… “Insect locomotion on solid surfaces spans a whole behavioral continuum (refs) – from slow walking to running, exploratory stepping, gap crossing, climbing, and turning.” I don’t agree with using the word “walking” twice in the first sentence here with two different meanings – walking as a slow stepping gait, vs. walking as a general term covering all terrestrial locomotion.

Thank you for this clarification, we followed your suggestion and tried to stick with the distinction.

REVIEWER 5 RESPONSE:

Reviewer #5: General comments

The authors describe an artificial neural network controlling different walking patterns of a hexapod with 18 DoF. The structure of the neural control system includes local rules coupling different legs through sensory feedback loops; no central oscillatory network setting a common locomotor rhythm and pattern is included in the network. Computer simulations demonstrated a wide range of possible locomotor patterns generated by the robot. The author concluded that such a decentralized control based on the local inter-leg connections via sensory feedback rules without a CPG are sufficient to generate walking for almost the entire range of walking speeds, except very fast ones.

The manuscript nicely fits the scope of the journal and is of interest to a wide range of researchers in the field of locomotor control of biological and robotic systems. The manuscript is well written with detailed rationale for the study, description of the relevant methods, results and discussion. Although the authors’ conclusion that the proposed neural network is capable of producing hexapod various walking patterns appears well justified, their claim that the same general neural network architecture might underlay locomotor control in legged animals is less convincing. The novelty of the proposed idea of decentralized locomotor control seems to be questionable. The literature review in the manuscript seem missing important relevant publications that might challenge authors’ interpretation and conclusions. These and other weaknesses detailed below need to be addressed.

We want to thank the reviewer for his suggestions and tried to address the raised issues. The Reviewer addresses three main critical points:

First, he points out that the parallel to “animals in general is less convincing”. We tried to clarify this in the revision. We agree with the reviewer and it was not our intend to draw this bold conclusion. Our main focus is on insects and to some extent to crustaceans. (now in lines 108 – 110) We only tried to point out a possible connection towards other locomotion approaches in more general which often draw the parallel to insect walking themselves. We tried to clarify and limit this to a brief remark in the introduction and one in the discussion.

Secondly, “novelty of decentralized control is questionable”: We will address this in more detail in below. In general, we don’t see the decentralized control as the novel contribution (in particular, as there are many predecessor models that originate from our lab). Instead, the novel contribution is the more detailed decentralized model (including intraleg coordination and a more biological neuron model) which is tested across a large variety of behavioral experiments and in particular applied in quite different experimental paradigms. We are not aware of another approach that is applying such a model in a realistic simulated system that includes sensory feedback and is tested in these kinds of neurophysiological experiments as well.

Last, the reviewer is pointing to missing important publications: concerning the latter point we are not quite sure what is meant. Probably, the Reviewer refers to mammal studies. They are however outside the scope of this work (see below).

Major comments

1. The claim that the proposed neural network structure can underlay locomotor control of legged animals (i.e., no CPG involvement for most of walking speeds or sensory feedback plays a subordinate role in studies proposing the main contribution of CPG) must be better justified or modified (lines 879-893). Such characterizations of studies supporting the CPG-based architecture and the role of sensory feedback in modulating CPG activity do not seem accurate. It has been well established since the 1920s-1930s by T. Graham Brown (see his unpublished videos of decerebrate walking cats: <https://www.youtube.com/watch?v=wPiLLplofYw>) and by Grillner with colleagues in 1970s-1980s (e.g. Forssberg et al. Acta Physiol Scand 108: 283-295, 1980) that the decerebrate and spinal cats can initiate and stop locomotion, change gaits from very slow stepping to galloping, or change locomotor rhythms differentially for the left and right hindlimbs based on sensory feedback exclusively. Similar changes in rhythmic activity patterns occur at different intensities of the locomotor region in midbrain of cat fictive locomotion preparations (no motion-dependent feedback). There are detailed CPG-based modes that predict gait changes from very slow walking to fast running based on a simple supraspinal velocity-related signal; the locomotor patterns and gait changes predicted by these models match well experimental recordings in genetically modified and wild type mice (e.g. Danner et al. Elife 6: 2017; Ausborn et al. Elife 8: 2019). In addition, fictive locomotion patterns (no motion-dependent sensory feedback) demonstrate a wide range of swing and stance durations covering a wide range of the corresponding walking speeds from very slow walking to fast running (e.g., Yakovenko et al. J Neurophysiol 94: 1057-1065, 2005).

The claim that neuroWalknet can underlay locomotor control in animals is questioned, because there are many data concerning mammals where CPG based modes control walking.

Response: we want to clarify that we don’t claim this. First we focus on insects and crustaceans only (now lines 108, 109). Second, we do not state that these animals do not use CPGs for control of walking. We however state that the currently available data may well be explained without requiring CPGs for the control of hexapod walking. And we furthermore state that even those data, which are often used to prove that CPGs do control insect walking for pentapod, tetrapod and tripod gaits, may be explained by a neuronal structure that is not equipped with CPGs when the insect is intact.

Reviewer 5 apparently interprets the text in lines 879 – 893 (now changed and in lines 1349 – 1363) as a statement, that we propose that also in mammals CPGs would not play a role for control of walking which is not what we intended. We quoted Clancy et al. simply as a possible connection to other animals as well, as Clancy and colleagues observe, for mammals, a tendency to focus on sensory feedback when controlling slow velocities. This is an interesting observation for discussion.

Already here we would like to mention that most of the arguments used by Reviewer 5 deal with mammals. However, as stated above, all these are not relevant for our approach.

2. The described neural network does not seem to have postural reflexes that would resist sudden perturbation of a leg or joint position. In biological legged animals, such muscle length reflexes change the feedback sign from resistive during postural tasks (exploration behaviors mentioned by the authors) to assistive reflexes during locomotion (e.g. Edward, Prilutsky, In: Neurobiology of Motor Control: Fundamental Concepts and New Directions, New York: Wiley, 2017). The proposed network always inhibits the antagonist activity by mutual antagonistic inhibition to avoid the resistive reflexes. This seems to be an important issue for a discussion in the manuscript. By the way, a CPG appears to offer an elegant solution to the reflex sign change between resting and locomotor conditions – the same central rhythmic command excites synergists and inhibit antagonists (e.g., Geertsenet et al. J Physiol 589: 119-134, 2011).

To explain the situation, we very briefly recall the structure of our network. First, we have to distinguish between states swing and stance. During swing there is a negative proportional position feedback, the output of which (error signal) represents, in principle, velocity controller setpoint of the final PID controller, whose output in turn provides the velocity signal to the motors. During stance the velocity set point (or reference value) is provided, in principle, by the ring net. We say “in principle”, because these set point signals are to some extent dependent on mutual inhibition or other inputs (e.g. levator reflex, or leg lifting during swing) or coordination influences. The PID controller is integrated in the motor controller.

Insects as well as neuroWalknet have postural reflexes: in our network there are resisting reflexes for height control (see Methods, now lines 1563 – 1596), during stance, in the form of negative feedback controllers in the beta joint, for example. Further, there is a reflex avoiding mechanical contact during swing which is implemented in our network, too. In other words, resistive reflexes are not avoided. This is discussed in more detail in Discussion, sect B, now lines 1111 – 1183.

Nonetheless, thank you for pointing us to the textbook article of Edward and Prilutsky, which helped to better understand your question: These authors describe and distinguish between resistive reflexes and assistive reflexes, often termed as negative feedback or positive feedback. However, there is an eventually observed misunderstanding. The sign (positive, negative) might either be used to refer to the sign by which the (e.g. sensory) feedback signal operates on the top-down input of the network, i.e. the value of the set point (or reference input). This is the terminology as used in systems theory, then called negative feedback or positive feedback. However, biologists sometimes call resistive output (“reflex”), when the (motor) output opposes the intended velocity, and name it assistive, if the (motor) output supports the intended velocity (e.g. retractor movement during forward walking and stance). However, the latter case might result from a – in the sense of systems theory – negative feedback controller. This can be the case if the set point might not be fixed, but moving over time (e.g. Fig. S2). Then, depending on setpoint value relative to the sensory input value, the effect of the network might be assistive or resistive, without any “reflex reversal” required on the neuronal level/structure.

Therefore, the terminology often used by neurobiologists, not only by Edward and Prilutsky, is from our point of view misleading. This is the reason why we do not use the terms assistive and resistive, but use “negative feedback”.

Another simple example: If there is a given set point for velocity, and due to friction the actual velocity is slower, the negative feedback tries to speed up the movement of the joint (assistive). If, say due to slipping, the joint is faster than the set point velocity, the same negative feedback would slow down the movement (resistive).

Explicit assistive reflexes are not yet implemented, i.e., networks where active weights from (sensory) input change from inhibitory synapses to excitatory ones and back.

3. The authors compare their computer simulations with recordings obtained in real hexapods. It is however not clear how differences in inertial leg properties between their simulated robot and real hexapods could affect the comparison. Inertia of real legs can be ignored (e.g. Hooper et al. J Neurosci 29: 4109-4119, 2009) but that of the robot, which is 20 times larger, probably not. Another difference on the design between the real hexapod and the robot is the presence of multi-joint muscles and passive tissues that couple leg joints; there are no similar structures in the robot.

It is of course true, that insect and robot show different inertial properties.

In insects leg inertia is small, near zero, but there are further complications that have to be taken into account: There is a body as well. More critical is friction, as mentioned (now lines 1886 – 1898), and most simulation studies based on simulated muscles did not consider body inertia at all, because they do not use a physical or dynamically simulated body/robot (one exception is Rubeo et al. 2018 as mentioned)

To compare the effects of inertia alone is quite difficult, because the behavior does not only depend on inertia, but also on the power of the motors (specially designed for this robot, Duerr et al. 2019) friction and the parameters of the PID-controller. As our goal is not to simulate exactly the temporal and velocity trajectories of the leg but the leg patterns in various behavioral situations, we tuned the parameters to reach appropriate swing and stance durations and their respective end positions. This was simplified by applying 10 fold slower velocity of the robot.

To study spatiotemporal patterns, the exact trajectory of the swing movement is not important, but the time between beginning and end of swing and stance is crucial. Nonetheless, we tried to roughly approximate the shape of swing trajectory to that of stick insects, but did not model the detailed velocity profile during swing.

Therefore, taken together, different inertia appears not critical in our case.

*Multi-joint muscles, passive tissue:* It is true that there are no multiple joint muscles in our robot, but a model requires to select a level of abstraction and what to include into a model. For our purpose detailed muscle models or models of muscles spanning multiple joints are not important. The questions addressed in our approach are not very much dependent on the detailed nature of simulating muscles. In our case muscles are simulated by elastic springs (i.e. passive tissue) plus nonlinearities (Methods, now lines 1477 – 1500, 1563 – 1574) and the amplification factor of the negative proportional position feedback loop for the beta joint (and a nonlinear height control, see also Fig S1).

4. The authors suggest that their distributed neural network model without CPG is novel. This claim may be inaccurate as somewhat similar models have been described in the literature and should be discussed in the manuscript (e.g. Geyer, Herr (2010) IEEE Trans.NeuralSyst.Rehabil.Eng. 18, 263–273; Dzeladini et al. Front Hum Neurosci 8: 371, 2014).

The reviewer is correct that distributed networks for hexapod without CPGs is not novel and we didn’t want to make this claim. Such networks are known since long including a list of papers from our own group beginning with Cruse (1980) followed by different earlier versions of Walknet as referenced in the article. Furthermore, this point is also discussed in detail by the excellent contributions of Bässler (1986) and Pearson (1987) that support this view and which we now have added to the paper (now lines 75, 337, 347, 1099), as recommended by Reviewers 2 and 3.

Actually, there are different definitions available concerning CPGs. Griller and Wallen (1985) now quoted on line 339 and 347, include that CPGs contribute to walking. Bässler (1986) in his excellent conceptual paper states that CPGs are only defined for deafferented neuronal systems. If oscillations are observed in such a preparation, this does however not mean that this CPG controls the walking pattern of an intact animal. Similarly, Pearson (1987) states that there is no evidence for contribution of CPGs to walking. Büschges et al. (1995) said that *“In summary, except for the anti-phase activity of the antagonistic excitatory motoneurones, no feature of the pilocarpine-induced rhythmicity […] appears to correspond to any motor output observed in the intact animal”* [ i.e. stick insect], Büschges, Schmitz, Bässler 1995, J exp Biol 198, 435-456.

From our point of view, the novel contributions are the following (we tried to clarify this in the paper):

(i) the neural version of Walknet (therefore neuroWalknet), and
(ii) inclusion of many more behaviors observed in insects (and crustaceans) including experiments with pilocarpine studies, i. e., a much larger number of biological data covered by one simulated network than has been observed before, which
(iii) includes the interpretation that CPGs may not be required to control insect walking plus several hypotheses concerning the embedding of specific known neurons,
(iv) by connecting neuroWalknet to a dynamically simulated robot to close the loop through the world.

Importantly, all what we describe here as novel is independent of the details how muscles are simulated or inertia value of Hector`s legs.

Specific comments

Line 22, “for starting or 22 interrupting a walk or changing velocity, all being difficult for CPG controlled solutions”: This statement does not seem accurate -- recent CPG models accurately predicts changing gaits with increasing locomotion speed (see Major comment 1 for references).

Line 22: For references given in major comment 1: As mentioned, we deal with insects. Here we quoted two very recent papers that underline this statement concerning starting and stopping. The situation might be different in mammals as they show quite different leg morphology. But we agree that “changing velocity” is not a serious problem for CPG driven systems. We have changed this sentence accordingly, thanks a lot.

Lines 68-69, “But most of the cases studied concern swimming or flying or fast running”: Can the authors provide references for this statement, which does not seem accurate. For example, Yakovenko et al (J Neurophysiol 94: 1057-1065, 2005) have demonstrated a wide range of flexor and extensor burst activities in the cat fictive locomotion preparation corresponding to very slow walking to fast running in the cat.

Now lines 78 – 80: Again, we do not deny this, but we refer to insects, not the cat.

“But most …” As we have not performed a quantitative search, we replaced “most” by “many”. Thanks a lot for pointing out this weakness.

Lines 139-141, “the fastest reflex response requires between 16 and 20 ms”: I understand that these numbers are taken from a published work, but the authors should keep in mind that this reflex response time is actually much longer than the fastest reflex in a much larger animal, the cat; the stretch reflex in cats has a delay of 8-10 ms.

Now lines 154 – 156: Yes, the data stem from cockroach studies, which are relevant for us as we deal with insects, not mammals. But even when addressing the short stretch reflex in cat: Note that insect neurons have much slower conduction velocities. On the other hand, insects may show quite short time delays (see Höltje and Hustert 2003), so it seems difficult to end up with definite conclusions at this point.

Line 272, “velocity signals”: velocity-dependent signals?

Now line 419; This sounds much better and we changed it – thanks. Now line 320

Lines 278-279, “The joints of a leg are often not moved in phase, but, depending on the current environmental situation, may show varying phase values.”: Inter-joint coordination can be affected by multi-joint muscles and their passive properties and sensory feedback. This aspect of coordination is not discussed but appears to be important at least in mammalian locomotion (cheetahs, horses, humans).

Line 278 – 279: Interjoint coordination in mammals: this might be true, but we deal with arthropods, see also our earlier response to multi-joint muscles.

Lines 336-338, “As an insect, the model has three pairs of legs, front legs, middle legs and hind legs…”: There is a critical difference in the inertial properties and thus dynamic behavior of robotic and insect legs -- the inertia of insect legs can be neglected (Hooper et al. 2009) but not in robot. It is important to discuss this difference and its effects on locomotor dynamics and control (see also Major comment 3).

Now lines 425 – 426: inertial properties: we have already addressed this point above (major point 3). In short: our robot is in fact 10 times slower, motor parameters and feedback parameters are tuned to produce leg movements sufficient to reach the goals addressed here.

Lines 384-390, “Using the traditional characterization…”: Which elements of the control structure prevents 2-, 1- and no limb support phases?

Now lines 552 – 560: Our decentralized structure does not control specific support phases. Therefore, no such structures are implemented to prevent these support phases. What we can say is that after very difficult starting configurations 2-limb support phases may occur, but if so, they are very short and stable walking will result after at least three steps (data not published, see line 849 - 856). This property is mainly due to the decentralized architecture. It is definitely a much smaller problem for hexapods compared to mammals.

Line 613, “a decentralized controller”: This term is somewhat misleading as the network has "central rules" of control that are applied to all DOF.

Now line 919: decentralized controller: These rules (addressed as “central rules” by Reviewer 5) only act between neighboring legs and contribute (in addition to local leg influences) to transition between swing state and stance state of an individual leg. The DoFs of a leg are controlled locally within the leg controller. The only central influence is given by walking velocity. Therefore, our controller is decentralized (i) on the level of the individual leg and (ii) on the level of coupling between legs (in contrast to approaches that use a central CPG to select various gaits, for example (e.g. Steingrube et al 2010).

Lines 615-616, “a detailed neuronal realization based on an antagonistic structure”: This organization appears to resemble the CPG organization based on the unit burst generators proposed by Grillner, 1981. It would be appropriate to reference this work and discuss the differences in organization between the neuroWalknet and unit burst generators.

Now lines 921 – 922: the antagonistic structure as such is given by the fact that there are antagonistic motor output and antagonistic sensory input as found in insects (for early simulation see e.g. Cruse 1980). The structure is basically different from that of Grillner (1981). There is no CPG in the network for (simulated) intact insects, but lateral inhibition and no endogenuous input (Bässler 1986). CPG properties are elicited only after application of (simulated) pilocarpine.

A very related question has been formulated by Reviewer 1 and we briefly repeat our answer here:

*Our argument that this model does not use an explicit CPG during walking depends on four different points as addressed in Methods (now lines 1908 – 1920). Importantly, the structure mentioned by the reviewer, is not active in normal walking behavior: In particular, it depends on a large enough input to both antagonistic units, termed “endogenous input” by Bässler (1986). In our case these inputs (shown in Fig 2, “pilo”) are set to zero in all simulations apart from those where (simulated) pilocarpine is applied. Therefore, when simulating the intact animal, this network cannot produce central oscillations, and therefore there are no functional CPGs in the intact animal. Rather, there is only antagonistic input that depends on antagonistic sensors.*

*Only if a crucial change is applied to the circuit – in biological terms, only if legs are deafferented and pilocarpine is given, – a situation quite different from that of an intact insect, the network properties are dramatically changed which then allows for oscillations (now lines 1904 – 1920).*

Lines 758-760, “Furthermore, as no CPGs are applied on this level…”: Mutually inhibitory connections between the antagonist neurons may be considered CPG components as in the unit burst generators (Grillner, 1981). So the statement that 'no CPGs are applied to this level' may be somewhat misleading.

Now lines 1113 – 1115: As above (response to major comment 4): there is lateral inhibition, but no CPGs as the network of the intact insect does not contain bilateral endogenous input represented here by simulated pilocarpine. Without pilocarpine it cannot oscillate. See also responses to Reviewer 1 as briefly repeated above.

Line 771, “Levator Reflex”: It is not clear from the model description and Discussion how this and other motor reflexes are regulated by the network to ensure that postural reflexes do not interfere with the ongoing locomotion (e.g. Edwards, Prilutsky In Neurobiology of Motor Control (eds. Hooper, Buschges), 2017.

Now line 1178: Levator reflex: the levator reflex does not directly interfere with the controller on the neuronal level (an example for decentralized architecture). Compensation is done indirectly: if, due to the reflex activation, swing duration is prolonged, this affects interleg and intraleg coordination via sensory input. The levator reflex was studied in detail by Schumm and Cruse (2006), but is not detailed here again, as it does not touch the main questions studied in the current paper.

Lines 795-797, “These results provide an alternative explanation…”: As mentioned in the previous comments, a CPG does not have to consist of a central network setting a single rhythm and pattern (e.g., Grillner, 1981). On the other hand, the idea of locomotor control without CPG is not completely novel either -- see for example Geyer, Herr (2010) IEEE Trans.NeuralSyst.Rehabil.Eng. 18, 263–273; Dzeladini et al. Front Hum Neurosci 8: 371, 2014). That should be acknowledged in Discussion.

Now lines 1194 – 1198: Novelty of locomotion without CPG: again, we deal with insects, and again, we do not state that locomotion without CPG is novel (see response to major comment 4 and “specific comments, line 615, 616 – now in lines 921 – 922), see also the papers from Bässler 1986 and Pearson 1987, as mentioned in the contribution of Reviewers 2 and 3.

Here we add another brief response to Reviewer 1 which is related to this point:

*Our question is not if there are CPGs to be found in an insect neural system, but if the CPGs observed when treated with pilocarpine are responsible for controlling walking movement. This is a quite difficult question when dealing with an animal (see the still very relevant excellent papers by Bässler, 1986, and Pearson, 1987). However, in a simulation study this is possible, because here the situation is very different as the case is clearly defined. It is known if a CPG is implemented and activated and what exactly is its function in the system.*

Line 808, “velocities of 10mV or less”: Sounds awkward’ consider changing e.g. to “Velocities corresponding to 10mV or less”.

Now line 1256: Thank you, that sounds much better.

Lines 865-868, “This suggests…”: In these situations the system also relies on the intrinsic stiffness properties of the legs to resist perturbations (e.g. Spagna et al. Bioinspir Biomim 2: 9-18, 2007).

Now lines 1335 – 1338: intrinsic stiffness properties: Yes, we completely agree. Therefore, on (the old) line 866, we had already set in brackets … (e.g. neural) pattern generators … to include possible mechanical structures. Due to a recommendation of Reviewer 3, we have now shortened this sentence, but intrinsic stiffness properties have already been addressed in the Intro at several places.

Lines 881-884, “For more natural behaviors…”: This characterization of the role of sensory feedback in CPG-based models does not seem accurate (Major comment 1).

Now lines 1351 – 1354: we deal with insects and their simulation and only at the very end refer to a study on mammals. We added “arthropod” or “insect” at several places to avoid misunderstandings.

Please apologize the repetitions in our answers. As mentioned, we focus on insects (and some extent to crustaceans) and we mention this early in the Introduction. We tried to clarify this further and in particular in the two remarks on possible connections. We tried to be clear that this is not meant as a generalization to animals in general.
