# Supplementary material for: Correction: Decentralized control of insect walking: A simple neural network explains a wide range of behavioral and neurophysiological results
Source: PLoS Comput Biol. 2021 Sep 2;17(9):e1009362. doi: 10.1371/journal.pcbi.1009362 (PMC8412292; doi:10.1371/journal.pcbi.1009362)
Supplement: S1 Document — (DOCX) [file pcbi.1009362.s001.docx]

Supporting Information

S1 PDF – **Motor Activation during Stepping Cycle**

in Malte Schilling and Holk Cruse (2020): Decentralized control of insect walking - a simple neural network explains a wide range of behavioral and neurophysiological results. *PLOS Computational Biology*





**Figure S1 PDF–1. Sections of steps for different legs.** A) Motor output provided by the six motor neurons (Fig 2, MN). B) Approximation of torque values provided by the six muscles for the same steps as in A (see text). Walking velocity 30 mV. Right legs, alpha joint: protractor green, retractor red; beta joint: levator green, depressor red; gamma joint: flexor green, extensor red. Each subfigure starts and ends with a swing phase highlighted by grey shadows. Abscissa: time (s) separate for each subfigure, ordinate (mV). Right ordinates show joint angle in rad (blue).

The figure shows motor output provided by the six motor neurons (Fig. 2, MN) and Fig 2SB shows an approximation to torque values provided by the six muscles of the three legs (front leg, middle leg, hind leg) during a complete step cycle. Sensory input required for performing a swing movement or a stance movement is given by the light grey units (Fig. 2). These units receive sensory input (position and load) from the own leg and from other legs via interleg coordination influences. Swing state, as represented by activation of the leftmost light grey unit in Fig. 2, is highlighted grey in the Fig. S1 PDF–1 above. Within each mode, motor units of the different joints are, during swing, controlled by negative feedback controllers, which, depending on the current leg position, may activate one of both antagonists. During swing, a HPF signal acts as a temporally limited disturbance signal at the beginning of swing thereby lifting the leg. Set points are fixed, but are different for different joints. During stance, the motor output of alpha joint antagonists and gamma joint antagonists receive signals via the ring net which depend on the current angle alpha (and the global walking direction). The beta joint is governed by a proportional negative feedback controller the set point of which depends on gamma angle, in order to maintain an about constant body – ground distance (see Supporting information Fig. S1 Fig). Depending on the somewhat different geometrical position of the legs (and the different position of the COM), the temporal order of antagonist activations is different for the different legs. Note that switches between antagonistic muscles can be found at different temporal moments during the complete cycle, even within swing mode or stance mode. As discussed by Dallmann et al. [1], motor output activity and torques may operate in opposite direction as is obvious in the second part of gamma joint of the hind leg or the first part of the gamma joint in the middle leg.

Simulation results depicted in Fig. S1 PDF–1 (B) may be compared with torques received from stance movements of free walking stick insects [1]. In all the legs, there is a good agreement with respect to the beta joint, where the depressor (red) provides the torque required to support the body. Qualitative agreement can be observed as well in the gamma joints. The extensor torque dominates the stance of the middle leg and the second part of the front leg in both biological results and simulation. The second part of the hind leg stance is characterized by flexor torque in both simulation and biological experiments, in spite of extensor velocity activation. Results differ in the first part of the front leg stance, where simulation shows extensor torque activation, whereas the biological experiments show flexor torque activation. In the first part of hind leg stance, simulation shows flexor activation whereas the biological experiments show extensor activation. Concerning the alpha joint, there is good agreement with respect to the front leg and the middle leg. The hind leg simulation shows an irregular alternation between protractor and retractor torque, as does the biological data. However, the tendencies are different. In the biological data retractor dominates in the first section of stance, and protractor in the second part, opposite to that found in the simulation.

Taken together, there is good overall agreement for middle leg and for the beta joint of front leg and hind leg, but no clear match in alpha joint and gamma joint of the front leg and the hind leg, where the biological data do not show a very clear picture either. Note that a perfect fit is not to be expected due to the difference of leg geometry and, importantly, different position of center of mass in both cases (see Methods).

**References**

1. Dallmann CJ, Dürr V, Schmitz J. Joint torques in a freely walking insect reveal distinct functions of leg joints in propulsion and posture control. Proc R Soc B. 2016;283:20151708.
