# Supplementary material for: Correction: Decentralized control of insect walking: A simple neural network explains a wide range of behavioral and neurophysiological results
Source: PLoS Comput Biol. 2021 Sep 2;17(9):e1009362. doi: 10.1371/journal.pcbi.1009362 (PMC8412292; doi:10.1371/journal.pcbi.1009362)
Supplement: S3 Document — (DOCX) [file pcbi.1009362.s003.docx]

Supporting Information

S3 PDF – Discussion: **Interleg Coordination**

in Malte Schilling and Holk Cruse (2020): Decentralized control of insect walking - a simple neural network explains a wide range of behavioral and neurophysiological results. *PLOS Computational Biology*

**Differences between species** concerning interleg coordination and chosen coordination influences

As to be expected, in spite of similar basic results observed in different species mentioned, there are some differences, too. In rock lobster, the ipsilateral in-phase influence from a walking leg onto a standing leg has only been observed to act in rearward direction [1], whereas in intact stick insects rule 5 influences are provided to both anterior and posterior neighboring legs. In Borgmann et al. [2] an anterior influence from a walking middle leg to a deafferented front leg has not been found. Mantziaris et al. [3] found no or weak contralateral coupling among front legs in *Carausius m.*, in contrast to locusts (legs deafferented,[4]) and stick insects walking on a treadmill [5,6]. These differences may be species specific or may depend on specific methodological properties, for example the concentration of pilocarpine or different friction of the treadmills.

A more general difference appears to exist between coordination rules found in insects (*Carausius* morosus [7]) and crustaceans (crayfish *Astacus leptodactylus* [8,9]). In the latter case coordination is stabilized by variation of swing duration instead of duration of stance. This difference has been assumed to result from basic differences concerning the environmental conditions [10].

**Coordination Influence 4:** Targeting of leg movements

Targeting is not addressed because it appears to be switched on and off depending on the current walking mode. Targeting seems to be switched off during negotiating of curves, but possibly switched on between front legs during tight turns and may be switched on between middle and hind leg during very tight turning (personal observations). Therefore, targeting behavior appears to be more complex than just operating from hind to middle legs or from middle to front leg as assumed to date. As Schilling et al. [11] have shown that an internal body model is well suited to support curve walking, one may speculate that the variability of targeting behavior might require some kind of body model instead of a number of separated modules. Data that support this view are given by Dürr and Schilling [12].

**References**

1. Clarac F, Chasserat C. Experimental modification of interlimb coordination during locomotion of a Crustacea. Neuroscience letters. 1979;12:271–6.

2. Borgmann A, Hooper SL, Büschges A. Sensory Feedback Induced by Front-Leg Stepping Entrains the Activity of Central Pattern Generators in Caudal Segments of the Stick Insect Walking System. The Journal of Neuroscience. 2009;29(9).

3. Mantziaris C, Bockemühl T, Holmes P, Borgmann A, Daun S, Büschges A. Intra- and intersegmental influences among central pattern generating networks in the walking system of the stick insect. J Neurophysiology. 2017;118:2296–2310.

4. Knebel D, Ayali A, Pflüger HJ, Rillich J. Rigidity and Flexibility: The Central Basis of Inter-Leg Coordination in the Locust. Front Neural Circuits. 2017;10:112.

5. Cruse H. Coactivating influences between neighbouring legs in walking insects. Journal of Experimental Biology. 1985;114:513 519.

6. Cruse H. A quantitative Model of Walking Incorporating Central and Peripheral Influences. I. The control of the individual leg. Biological Cybernetics. 1980;37:131–6.

7. Cruse H, Bläsing B, Dean J, Dürr V, Kindermann T, Schmitz J, et al. WalkNet - a decentralized architecture for the control of walking behaviour based on insect studies. In: Pfeiffer F ZT, editor. Walking: Biological and Technological Aspects CISM Courses and Lectures No 467. International Centre for Mechanical Sciences. Wien, New York: Springer-Verlag; 2004. p. 81–118.

8. Cruse H, Müller U. Two coupling mechanisms which determine the coordination of ipsilateral legs in the walking crayfish. Journal of Experimental Biology. 1986;121:349 369.

9. Müller U, Cruse H. The contralateral coordination of walking legs in the crayfish Astacus leptodactylus. I Experimental results Biol Cybern. 1991;64:429–436.

10. Cruse H. What mechanisms coordinate leg movement in walking arthropods? Trends in Neurosciences. 1990;13:15–21.

11. Schilling M, Paskarbeit J, Schmitz J, Schneider A, Cruse H. Grounding an Internal Body Model of a Hexapod Walker — Control of Curve Walking in a Biological Inspired Robot. In: inproceedings. 2012. p. 2762–2768.

12. Dürr V, Schilling M. Transfer of Spatial Contact Information Among Limbs and the Notion of Peripersonal Space in Insects. Front Comput Neurosci [Internet]. 2018 [cited 2019 Jul 30];12. Available from: https://www.frontiersin.org/articles/10.3389/fncom.2018.00101/full
